# Precisely tuneable energy transfer system using peptoid helix-based molecular scaffold

**DOI:** 10.1038/s41598-017-04727-0

**Published:** 2017-07-06

**Authors:** Boyeong Kang, Woojin Yang, Sebok Lee, Sudipto Mukherjee, Jonathan Forstater, Hanna Kim, Byoungsook Goh, Tae-Young Kim, Vincent A. Voelz, Yoonsoo Pang, Jiwon Seo

**Affiliations:** 1 Department of Chemistry, School of Physics and Chemistry, Gwangju Institute of Science and Technology, 123 Cheomdan-gwagiro, Buk-gu, Gwangju, 61005 South Korea; 2 Department of Chemistry, Temple University, 1901 N. 13th St., Philadelphia, PA 19122 USA; 3 School of Earth Sciences and Engineering, Gwangju Institute of Science and Technology, 123 Cheomdan-gwagiro, Buk-gu, Gwangju, 61005 South Korea

## Abstract

The energy flow during natural photosynthesis is controlled by maintaining the spatial arrangement of pigments, employing helices as scaffolds. In this study, we have developed porphyrin-peptoid (pigment-helix) conjugates (PPCs) that can modulate the donor-acceptor energy transfer efficiency with exceptional precision by controlling the relative distance and orientation of the two pigments. Five donor-acceptor molecular dyads were constructed using zinc porphyrin and free base porphyrin (Zn(*i* + 2)–Zn(*i* + 6)), and highly efficient energy transfer was demonstrated with estimated efficiencies ranging from 92% to 96% measured by static fluorescence emission in CH_2_Cl_2_ and from 96.3% to 97.6% using femtosecond transient absorption measurements in toluene, depending on the relative spatial arrangement of the donor-acceptor pairs. Our results suggest that the remarkable precision and tunability exhibited by nature can be achieved by mimicking the design principles of natural photosynthetic proteins.

## Introduction

In natural photosynthetic proteins, the array of chlorophylls plays an essential role in the light-harvesting process, and in transferring the harvested energy to the reaction center (RC). Adjacent chlorophylls, which are precisely arranged in light-harvesting complexes (LHCs) at a distance of ~10 Å, interact strongly with, and are energetically coupled to each other, thereby delocalizing their excited states^1, 2^. Inspired by natural proteins, numerous attempts have been made to organize porphyrins, the core chromophores of chlorophylls, in higher order structures by displaying them on other scaffolding materials such as peptides^3, 4^, synthetic polymers^5–7^, DNA^8–11^, and even viruses and bacteriophages^12–15^. In addition to mimicking the natural light-harvesting system, porphyrin arrays have been extensively investigated for their potential use as sensors^16–18^, catalysts^19^, and photodynamic therapy agents^20^.

However, construction of a non-covalently interacting porphyrin array with a precisely defined set of distances and orientations has been hampered by the lack of efficient scaffolding materials and the difficulty in positioning individual porphyrins at will. As a result, studies of through-space interactions among porphyrins in defined distances and orientations, especially in face-to-face arrangements, have been rarely achieved and still remained as a challenge. The development of efficient artificial LHCs to further elucidate natural photosynthetic systems necessitates the identification of efficient scaffolding materials to precisely control the interactions among porphyrins with synthetic accessibility.

Peptoids are a class of bio-inspired peptide derivatives based on oligo-*N*-substituted glycines. Peptoids have emerged as attractive functional materials since the discovery of the submonomer protocol^21^, primarily because of their synthetic versatility and side chain diversity (arising from the variability in available primary amine submonomers). Sequential addition of monomers by solid-phase synthesis enables chemists to control the chain length, side chain functionality, and monomer sequence of desired peptoids. Post-synthetic decoration of peptoid side chains with various functional moieties is also well established^22^. Unlike natural peptides, peptoids are devoid of backbone hydrogen bond donors; however, a well-defined helical structure can be generated by steric and electronic interactions between the backbone amides and α-chiral aromatic side chains^23–26^. The conformations of canonical peptoid helices have a periodicity of three residues per turn and a pitch length of ~6.0 Å, resembling natural poly-proline type I (PPI) structures^25, 27–29^. As a result, side chain modifications at desired residues with defined spatial distances and orientations are possible using peptoid helices. With these unique features, peptoids stand out as useful scaffolding materials that can be used to display functional groups at specific positions.

**Figure 1:**
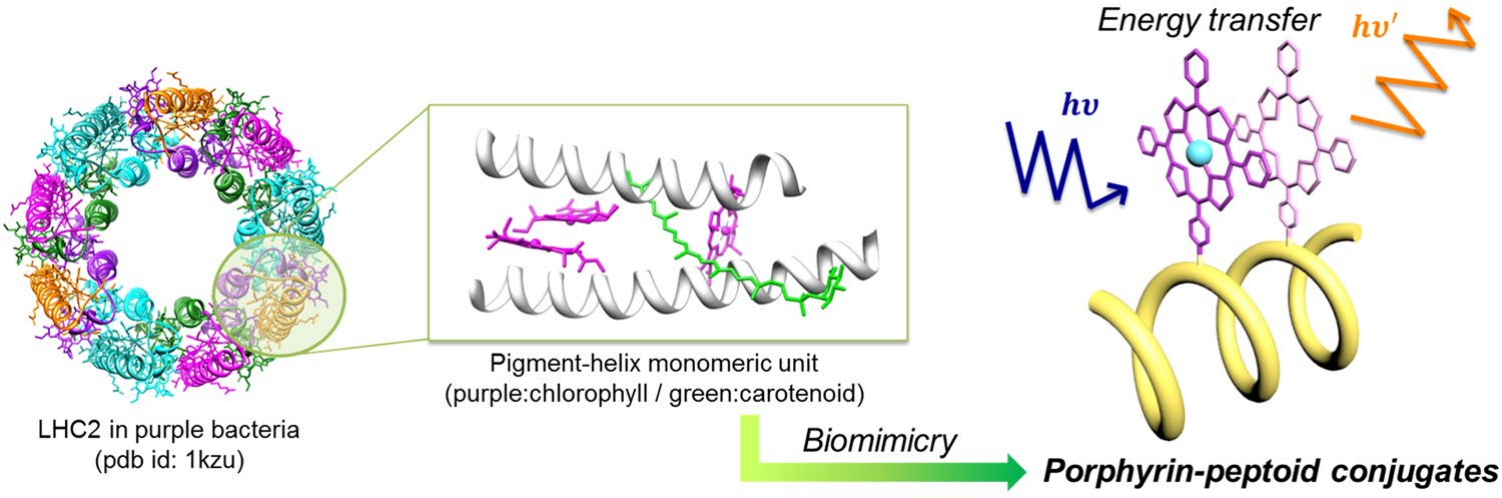
Light-harvesting complex II in purple bacteria and a single constituent pigment-helix unit.

Nature utilizes α-helical peptides and core chromophores as building blocks to construct large photosynthetic proteins (Fig. 1)^30^. Likewise, an artificial LHC can be constructed using α-helical peptoids and porphyrins as building blocks, resulting in a complex resembling a single unit of natural LHC. Previously, a set of porphyrin-peptoid conjugates (PPCs) with varying distances, orientations, and number of conjugated porphyrins, were synthesized taking advantage of the structural characteristics of peptoid helices^31^. Each PPC showed a distinct degree of J-aggregation via inter-porphyrin π-π interactions, depending on the spatial arrangement of the displayed porphyrins. In addition, dipole-dipole interactions between the displayed porphyrins were investigated with respect to the controlled spatial factors.

In the present study, we synthesized a set of molecular dyads comprising donors and acceptors. In the peptoid helix, the zinc-containing porphyrin was positioned as an energy donor, and the energy-accepting free base porphyrin was positioned with precise control of spatial relationship between the donor and the acceptor; consequently, these structures exhibited finely tunable and highly efficient energy transfer demonstrated by both static fluorescence emission and time-resolved transient absorption spectroscopy. The structural properties of porphyrin-peptoid conjugates (PPCs) were investigated with circular dichroism spectroscopy and molecular dynamics simulation. To our knowledge, this level of structural precision between the energy donor and acceptor has rarely been reported. Moreover, our donor-acceptor dyad system provides, (1) an example of the fine tuning of the energy transfer efficiency that is routinely carried out by nature, (2) a molecular platform that contributes towards better understanding of the physical chemistry of natural photosynthetic systems, and (3) a strategy for the modulation of porphyrin interactions that can be used in various applications, such as catalysis, sensors, and biomedicine.

## Experimental Section

### General methods

All reagents and solvents were purchased from Sigma Aldrich, Acros organics, Novabiochem and TCI. They were used without further purification. *N,N’*-Diisopropylcarbodiimide was purchased from Advanced ChemTech, KY, USA. For peptoid synthesis and conjugation reactions, peptide synthesis grade DMF and anhydrous DCM (>99.8%) were used, respectively. Flash column chromatography was conducted with silica gel 60 (230–400 mesh, Merck, Darmstadt, Germany) while monitored by thin-layer chromatography with precoated aluminum-backed TLC sheets (silica gel 60 F254, EMD Millipore, Billerica, MA, USA). For detection of protected amine, 2% ninhydrin in ethanol was used. Solvents were evaporated by rotary evaporator under reduced pressure. Abbreviations for reagents are as follows: 9-fluorenylmethoxycarbonyl (Fmoc); trifluoroacetic acid (TFA); triisopropylsilane (TIS); dichloromethane (DCM); *N*,*N*’-dimethylformamide (DMF); *N*,*N*’-diisopropylcarbodiimide (DIC); acetonitrile (ACN); chloroform (CHCl_3_); and 1-methyl-2-pyrrolidinone (NMP).

UV-vis spectra were measured on an Ultrospec 2100 pro UV-vis Spectrophotometer (GE healthcare, Buckinghamshire, UK) at room temperature in a quartz cuvette with 1 mm path length. HPLC grade DCM was used for a serial dilution of sample solutions and for a blank. Absorbance was observed in the range of 300–700 nm.

Fluorescence excitation and emission spectra of Zn PPCs were observed on an LS55 fluorescence spectrometer (PerkinElmer, Waltham, MA, USA) controlled by an FL winlab software. Typically, samples were prepared from a serial dilution with DCM. Emission measurements of Zn PPCs were performed with excitation wavelength at 550 nm, 12.0 nm slit width and 50 nm/min scanning speed. Fluorescence excitation spectra of Zn PPCs were obtained using the same samples with the conditions of 8.0 nm slit width, 200 nm/min scanning speed and two different emission wavelengths: ZnTPP specific emission wavelength at 605 nm and fbTPP specific emission wavelength at 716 nm. For negligible self-quenching, absorbance of Zn PPCs was kept below 0.1.

### Peptoid amine submonomers

**Figure 2:**
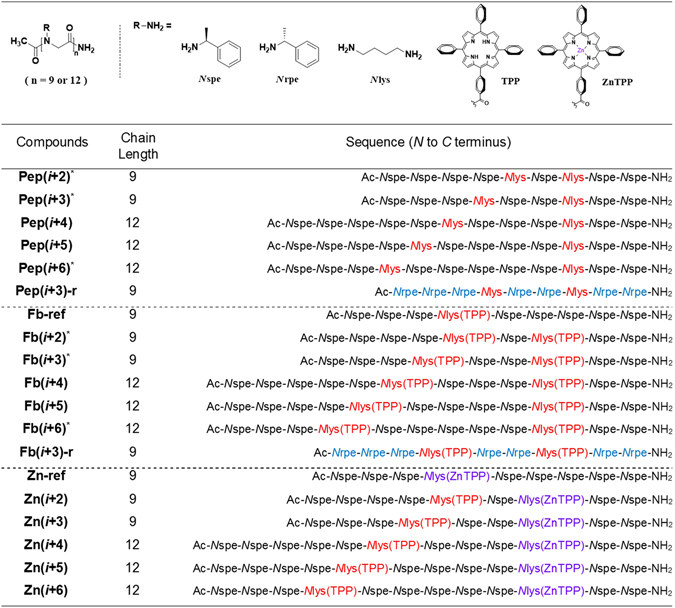
Sequences of Fb and Zn PPCs and their control peptoids (**Pep(** ***i + n*** **)**). Two porphyrins are n residues apart for a compound designated as (*i* + n). Opposite helix-sense is denoted by an ‘r’ at the end of the compound name, indicating that the helix-inducing monomers have an R configuration. In other words, **Fb(i + 3)** and **Fb(i + 3)-r** are enantiomers. The positions of fbTPP and ZnTPP at each PPC are highlighted. ^*^Six compounds that were previously reported are included for comparison^31^.

(*S*)-*N*-(1-Phenylethyl)glycine (or *N*spe, (*S*)-1-phenylethylamine) and (*R*)-*N*-(1-phenylethyl)glycine (or *N*rpe, (*R*)-1-phenylethylamine) were obtained from Sigma Aldrich (Milwaukee, WI, USA) at a purity of >99%. *N*-*tert*-Butoxycarbonyl-1,4-diaminobutane (or *N*lys(Boc)) and *para*-methoxytrityl-1,4-diaminobutane (or *N*lys(mmt)) were synthesized using a reported procedure^32^. Structures of the peptoid side chains derived from these amine submonomers are shown in Fig. 2.

### General procedure for peptoid synthesis

Peptoid nonamers and dodecamers were synthesized using microwave-assisted solid-phase submonomer synthesis methods^21^ on an Fmoc-Rink amide MBHA resin. A CEM MARS multimodal microwave reactor equipped with a fiber-optics temperature probe and a magnetic stirrer was used (CEM Corp., Matthews, NC, USA). The fiber-optics temperature probe was positioned in the reaction mixture, and the solution was stirred and irradiated at different reaction conditions as described below. All microwave reactions were carried out at atmospheric pressure. Fmoc-Rink amide MBHA resin (0.59 mmol/g, Novabiochem, San Diego, CA, USA) was used to generate *C*-terminal amide peptoids. After Fmoc deprotection, each monomer was added by a series of bromoacetylation and displacement of bromide by a primary amine^21^. These two steps were iterated with appropriate primary amines until desired peptoid sequence was obtained. The *N*-terminal acetylation of peptoid oligomers was performed by adding excess amount of acetic anhydride (50 equivalents) and pyridine (55 equivalents) in DMF. Typically, 0.25 mmol reaction scale was used (0.42 g of resin). The resin swelled in DMF for 20 minutes and drained. For Fmoc deprotection, the resin was treated twice with 20% (v/v) piperidine in DMF (5 ml each) at room temperature for 60 seconds and at 80 °C (microwave, 600 W max power, ramp 2 minutes) for 2 minutes. For bromoacetylation, bromoacetic acid (4.18 ml of 1.2 M bromoacetic acid in DMF stock solution, 5 mmol) and *N,N’*-diisopropylcarbodiimide (0.63 g, 5 mmol, 0.78 ml) were added, and the reaction mixture was stirred and irradiated at 35 °C (microwave, 400 W 15% power, ramp 0.5 minutes) for 2 minutes. For the displacement step, (*S*)-*N*-(1-phenylethyl)glycine (*N*spe, 5 ml of 2.0 M in NMP stock solution, 10 mmol), mono-Mmt protected 1,4-diaminobutane (*N*Lys(Mmt), 5 ml of 1.0 M in NMP stock solution, 5 mmol) or mono *tert*-butyloxycarbonyl protected 1,4-diaminobutane (NLys(Boc), 5 ml of 1.0 M in NMP stock solution) were added. The mixture was stirred and irradiated at 80 °C (microwave, 400 W 75% power, ramp 2 minutes) for 1.5 minutes. Between each step, the resin was thoroughly washed with DMF and DCM. The *N*-terminal acetylation was performed by adding acetic anhydride (1.28 g, 12.5 mmol, 1.2 ml) and pyridine (1.08 g, 13.7 mmol, 1.1 ml) to the resin-bound peptoid in DMF (1.5 ml). The reaction was stirred at room temperature for 2 h.

### Porphyrin conjugation

**Figure 3:**
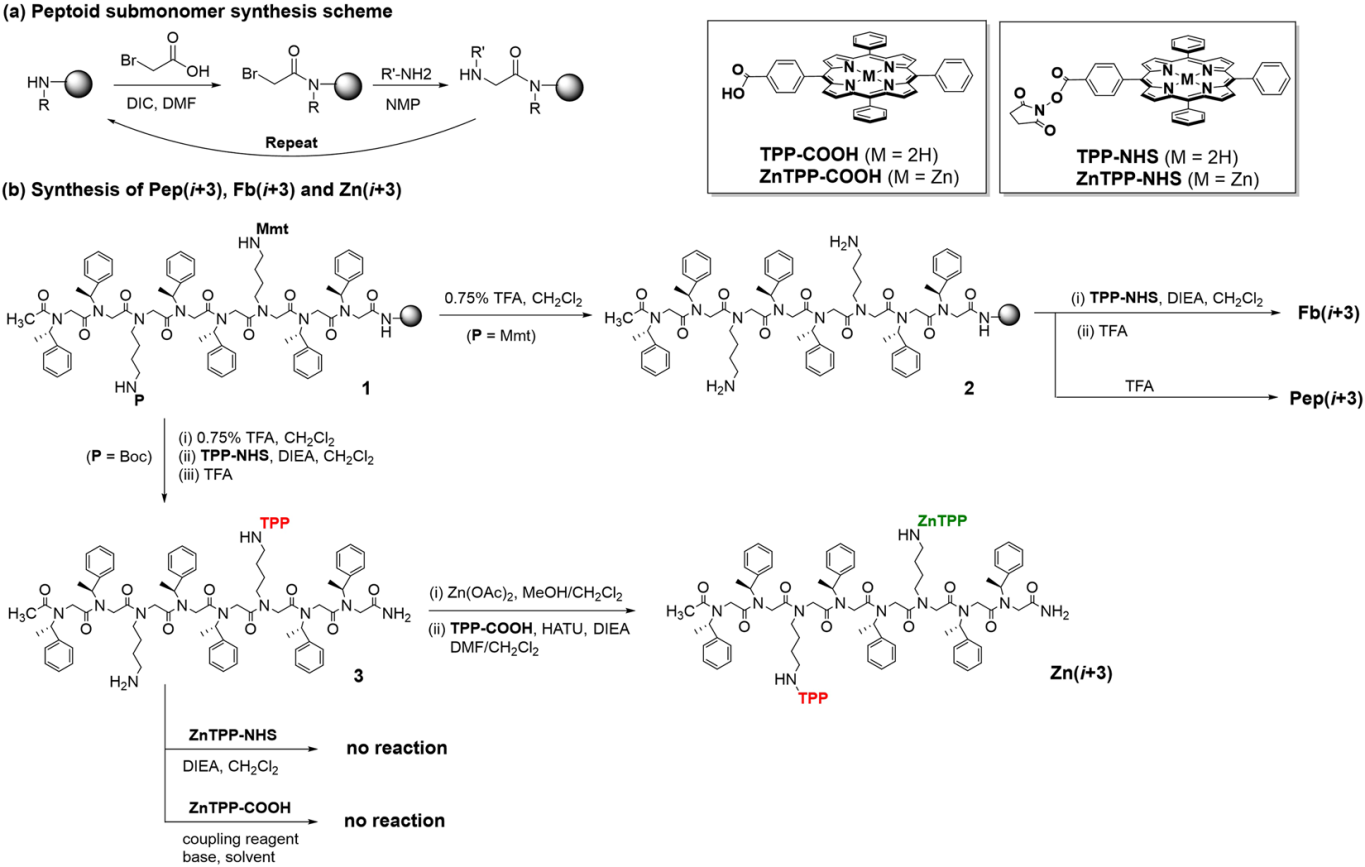
Synthesis of **Pep(** ***i*** **+** ***n*** **)**, **Fb(** ***i*** **+** ***n*** **)**, and **Zn(** ***i*** **+** ***n*** **)**. The synthesis of (*i* + 3) peptoids is shown for representative purposes.

Prior to porphyrin conjugation reaction, Mmt deprotection was performed by treating resin-bound peptoid with 0.75% TFA (DCM: TFA: TIS = 94.25: 0.75: 5). To the resin was added 0.75% TFA solution (6 ml), and stirring continued for 2 minutes at room temperature. After draining orange color solution, the resin was washed with DCM. These steps were repeated for 7 times or more until the drained solution became clear. Then, the deprotected amine was conjugated with fbTPP using the previously prepared fbTPP-NHS ester (See Supplementary Fig. S1). Typically, 1.5 equivalents of fbTPP-NHS ester per amine was used. Resin-bound and Mmt deprotected peptoid (0.0625 mmol) was washed first with the solution of DCM (4 ml) and DIEA (0.15 ml) for 1 minute to remove any residual TFA. To the resin was added fbTPP-NHS ester (150 mg, 0.20 mmol) in DCM (7 ml), followed by DIEA (0.07 ml, 0.40 mmol). The cartridge was sealed, and the reaction was stirred overnight under N_2_ atmosphere. The reaction mixture was drained, and the resin was washed thoroughly with DMF and DCM. For Zn PPCs, peptoid was then cleaved from resin using 95% TFA solution (TFA: DCM = 95: 5) for 13 minutes providing crude **3** (Fig. 3). The cleavage solution was filtered by solid-phase extraction (SPE) cartridges with 20 μ PE frit (Applied Separations, Allentown, PA, USA) and the volatiles were removed by lyophilizer. The crude peptoids were dissolved in ACN and purified by preparative HPLC (see HPLC and ESI-MS conditions in the following section).

The purified PPC (**3** in Fig. 3) (10.9 mg, 0.005 mmol) was dissolved in DCM (2 ml). To this solution was added diisopropylethylamine (DIEA, 5 equivalents, 3.4 mg, 0.026 mmol), and stirring continued for 10 minutes for deprotonation of porphyrin. TFA salts were removed by aqueous extraction, and the organic phase was collected, dried by adding Na_2_SO_4_, and concentrated. Compound **3** was dissolved in DCM (2 ml) followed by addition of zinc acetate (10 equivalents, 9.5 mg, 0.052 mmol) in MeOH (500 µl). The reaction mixture was kept stirring overnight. Removal of excess zinc acetate was performed by water extraction.

To the solution of peptoid with one ZnTPP in DCM (2 ml), DIEA (2 equivalents, 0.010 mmol) was firstly added. fbTPP-COOH (5-(4-carboxyphenyl)-10, 15, 20- tetraphenylporphyrin, 1.2 equivalents, 0.0063 mmol) and DIEA (1.2 equivalents, 0.0063 mmol) in DCM (1 ml) was added into the PPC solution followed by the addition of HATU (1.2 equivalents, 0.0063 mmol) in DMF (50 µl). The reaction mixture was kept stirring for overnight. After water extraction, desired product was purified using preparative HPLC.

### Purification of Zn(*i* + *n*) compounds

Analytical HPLC was performed on a Waters HPLC system (Waters 2489 UV-Visible Detector, Waters 1525 Binary HPLC Pump, Waters 2707 Autosampler, and Waters 5CH column oven) with a C18 column (SunFire C18, 4.6 × 250 mm, 5 *μ*m). The column oven temperature was set at 40 °C. Typically, the mobile phase was used as follows: (A, water + 0.1% TFA; B, ACN + 0.1% TFA) 2 minutes using 30% of B, a linear gradient to 100% B over 20 minutes, and then holding 100% B over 20 minutes. The flow rate was 1 mL/min. The purity of sample was monitored by absorbance at 220 nm. For **Zn-ref** and **Fb-ref**, the mobile phase was given as follows: 2 minutes using 50% of B, a linear gradient to 100% B over 15 minutes, and then holding 100% B over 30 minutes. Peptoids were purified by preparative HPLC system (Waters prepLC system, Waters 2489 UV-Visible Detector, Waters fraction collector III) with a C18 column (SunFire C18, 19 × 150 mm, 5 *μ*m) at a flow rate of 14 mL/min. For compound **3**, the mobile phase conditions were as follows: (A, water + 0.1% TFA; B, ACN + 0.1% TFA) 5 minutes using 50% of B, a linear gradient to 100% B over 20 minutes, and then holding 100% B over 15 minutes. For Zn PPCs, the mobile phase gradient for 60 minutes was given as follows: 10 minutes using 50% of B, a linear gradient to 80% B over 10 minutes and then holding over 15 minutes, a linear gradient to 100% B over 15 minutes and then holding over 10 minutes. Sample elution was monitored at 220 and 254 nm by absorbance. The purity of the product fractions was confirmed by analytical HPLC. Each fraction was further analyzed by LC/MS performed on an Agilent 6520 Q-TOF mass spectrometer. Additional SPE purification was performed to remove residual TFA (See Supplementary information, page 3).

## Result and Discussion

### Synthesis of Porphyrin-Peptoid Conjugates (PPCs)

A set of porphyrin-peptoid conjugates displaying two free base porphyrins (Fb PPCs) or one zinc porphyrin and one free base porphyrin (Zn PPCs) with various spatial arrangements of porphyrins were designed and synthesized employing the unique structural features of peptoid helices (three residues per turn periodicity and 6 Å pitch distance). The exact sequences of all PPCs are shown in Fig. 2. Nonameric peptoid scaffold was used for (*i*, *i* + 2), (*i*, *i* + 3), and reference PPCs, and dodecameric peptoid was used for (*i*, *i* + 4), (*i*, *i* + 5) and (*i*, *i* + 6) PPCs. Figure 3 shows the representative synthetic routes for Fb PPCs and Zn PPCs. Peptoid helices were synthesized employing a solid-phase and microwave-assisted submonomer protocol^21^. The helical folds were induced by an α-chiral side chain (*N*spe or (S)-(–)-1-phenylethylamine), which induces right-handedness^26, 27^. *N*lys (1,4-diaminobutane) was incorporated at the position of porphyrin conjugation. The synthesis of **Zn(** ***i*** **+** ***n*** **)** PPCs was not straightforward, primarily due to the lability of zinc porphyrin under acidic conditions; in other words, demetalation of zinc readily occurs under acidic conditions, such as exposure to TFA. Free base tetraphenylporphyrin (fbTPP) and zinc-containing tetraphenylporphyrin (ZnTPP) were located in the peptoid sequence at the position of *N*lys(Boc) and *N*lys(Mmt), respectively. After numerous attempts, metalation of **2**, followed by fbTPP conjugation appeared to be most feasible synthetic route that yielded a good quantity of the desired pure **Zn(** ***i*** **+ 3)**.

### Structural Analysis of Porphyrin-Peptoid Conjugates (PPCs)

**Figure 4:**
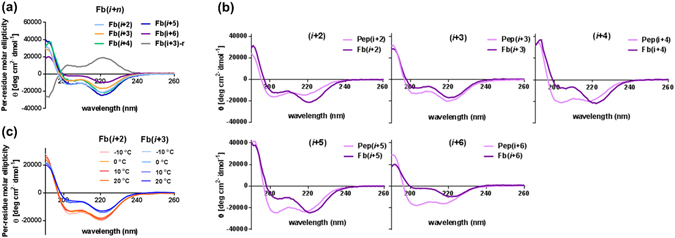
(**a**) Circular dichroism spectra of Fb PPCs recorded at 190–260 nm (50 μM in ACN). (**b**) Changes in CD spectra upon porphyrin conjugation: Prominent red shift was observed in all Fb PPCs (except **Fb(** ***i*** **+ 3)**) relative to their control peptoids, **Pep(** ***i*** **+** ***n*** **)**. Spectra of **(** ***i*** **+ 2)**, **(** ***i*** **+ 3)**, and **(** ***i*** **+ 6)**, which were reported previously, are shown for comparison^31^. (**c**) Temperature-dependent CD spectra of **Fb(** ***i*** **+ 2)** and **Fb(** ***i*** **+ 3)**.

The helicity of each Fb PPC was measured by circular dichroism (CD) spectra. It is generally known that the α-chiral and aromatic side chains of a peptoid sequence are strong driving forces for the development of polyproline type-I (PPI)-like helices, through steric and electronic interactions^23–26, 33^. PPCs are composed of monomers with α-chiral and aromatic side chains (such as *N*spe and *N*rpe); therefore, typical PPI-like CD signatures, with two minima at 202 nm and 220 nm and one maximum at 192 nm, were observed for all Fb PPCs. **Fb(** ***i*** **+ 3)-r**, designed with a monomer having the opposite chirality, showed a mirror image of **Fb(** ***i*** **+ 3)** (Fig. 4(a)).

The influence of porphyrin conjugation on the helical integrity of peptoids was also monitored. Differing propensities were observed based on the spatial arrangements of the displayed porphyrins. The helicity of the PPCs decreased when two porphyrins were displayed on the same face (such as **Fb(** ***i*** **+ 3)** and **Fb(** ***i*** **+ 6)**); the decrease in helicity was greater in the dodecamer (**Fb(** ***i*** **+ 6)**), compared to the nonamer (**Fb(** ***i*** **+ 3)**) (Fig. 4(b)). In other words, the population of PPI-type helical conformers reduced upon porphyrin conjugation. The 220 nm peak in the CD spectrum is known to reflect the contribution of *cis*-amide containing conformers; therefore, peptoid helical folds can be examined based on the intensity of this peak^34^. Interestingly, when two porphyrins were displayed at different faces (**Fb(** ***i*** **+ 2)**, **Fb(** ***i*** **+ 4)** and **Fb(** ***i*** **+ 5)**), the CD intensity at 220 nm increased, indicating that the population of the PPI-type helical conformers increased upon porphyrin conjugation. A noticeable red-shift was observed at ~ 220 nm in all PPCs after porphyrin conjugation, caused by the n → π* transition of carbonyl lone pair to π* orbital in the aromatic side chains^16^, except in **Fb(** ***i*** **+ 3)** (Fig. 4(b)). These results suggest the possibility of interaction between porphyrins and the adjacent aromatic side chains. Additional electronic interactions with porphyrins would stabilize the π* orbitals of side-chain phenyl groups, causing the red-shift of the 220-nm peaks. Porphyrins on **Fb(** ***i*** **+ 3)** might not have enough space to interact with phenyl groups, because of their spatial location next to each other at a distance of ~6 Å.

Along with the red-shifted CD spectra, the propensity of peptoid helical folds upon porphyrin conjugation could be interpreted based on the flexibility of the linker that connects conjugated porphyrins to the peptoid backbone. Since porphyrins are connected with flexible *n*-butyl linkers, they can move or rotate into a favorable conformation exhibiting minimum energy. For PPC displaying two porphyrins on a slipped-cofacial arrangement, porphyrins would bend to interact with each other in the direction of peptoid helical folds, as greater amount of energy (induced by steric and electronic interactions between chiral side chains) is required to resist the peptoid helical folds. As a result, movement of porphyrins towards the peptoid helical folds may result in strengthened helicity. On the other hand, PPCs displaying porphyrins on the same face do not need to move along with the peptoid helical folds to interact with each other. Therefore, non-directional movement of each porphyrin that occupies a significant part in the entire molecular weight of PPC may decrease the helical folds of peptoids. A comparison of **Fb(** ***i*** **+ 3)** and **Fb(** ***i*** **+ 6)**, which were composed of porphyrins arranged face-to-face, showed that porphyrins on **Fb(** ***i*** **+ 6)** had enough space to move, whereas porphyrins on **Fb(** ***i*** **+ 3)** did not; this was also revealed by a red-shift of 220 nm peak in the CD spectra. In addition, the larger heterogeneity of dodecameric peptoids relative to nonameric peptoids explains more significant effects on porphyrin conjugation in **Fb(** ***i*** **+ 6)**. Taken together, these results support the influence of relative porphyrin orientations on the maintenance of peptoid helical folds. In addition, as reported by Barron *et al*.^35^, we observed the peptoid CD signature of **Fb(** ***i*** **+ 2)** and **Fb(** ***i*** **+ 3)** did not differ significantly with variations in the temperature (Fig. 4(c)).

### Measurement of Energy Transfer Efficiency Using Absorption and Fluorescence Spectroscopic Analysis

**Figure 5:**
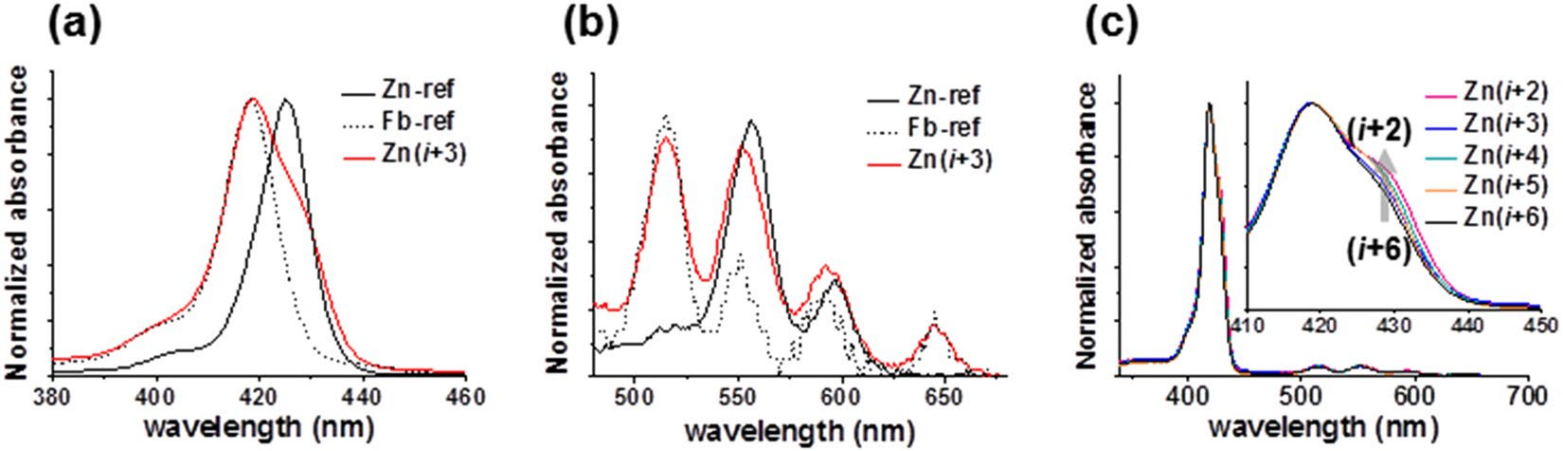
UV-vis absorbance of **Zn(** ***i*** **+ 3)** in (**a**) Soret band and (**b**) Q-bands, compared to **Zn-ref** and **Fb-ref**. (**c**) Absorbance of Zn PPCs depending on the spatial arrangement of porphyrins.

Spectroscopic analysis of Zn PPCs was performed in dichloromethane to avoid solvent (e.g., acetonitrile) coordination with Zn. In the Soret region, the absorption profiles of Zn PPCs were shown as an average of **Zn-ref** and **Fb-ref**, thereby verifying the existence of fbTPP and ZnTPP in Zn PPCs (Fig. 5(a)). In the Q-band region, **Zn-ref** showed two bands, because of the *D*_4*h*_ symmetry of zinc porphyrin, whereas both **Zn(** ***i*** **+ 3)** and **Fb-ref** showed four peaks (Fig. 5(b)). Interestingly, the degree of shift in the shoulder for Zn PPCs was slightly different depending on the spatial arrangement of the displayed porphyrins (Fig. 5(c), inset), with the shoulder intensity increasing around 430 nm from **Zn(** ***i*** **+ 6)** to **Zn(** ***i*** **+ 2)**, suggesting strongest donor-acceptor coupling in **Zn(** ***i*** **+ 2)**.

**Figure 6:**
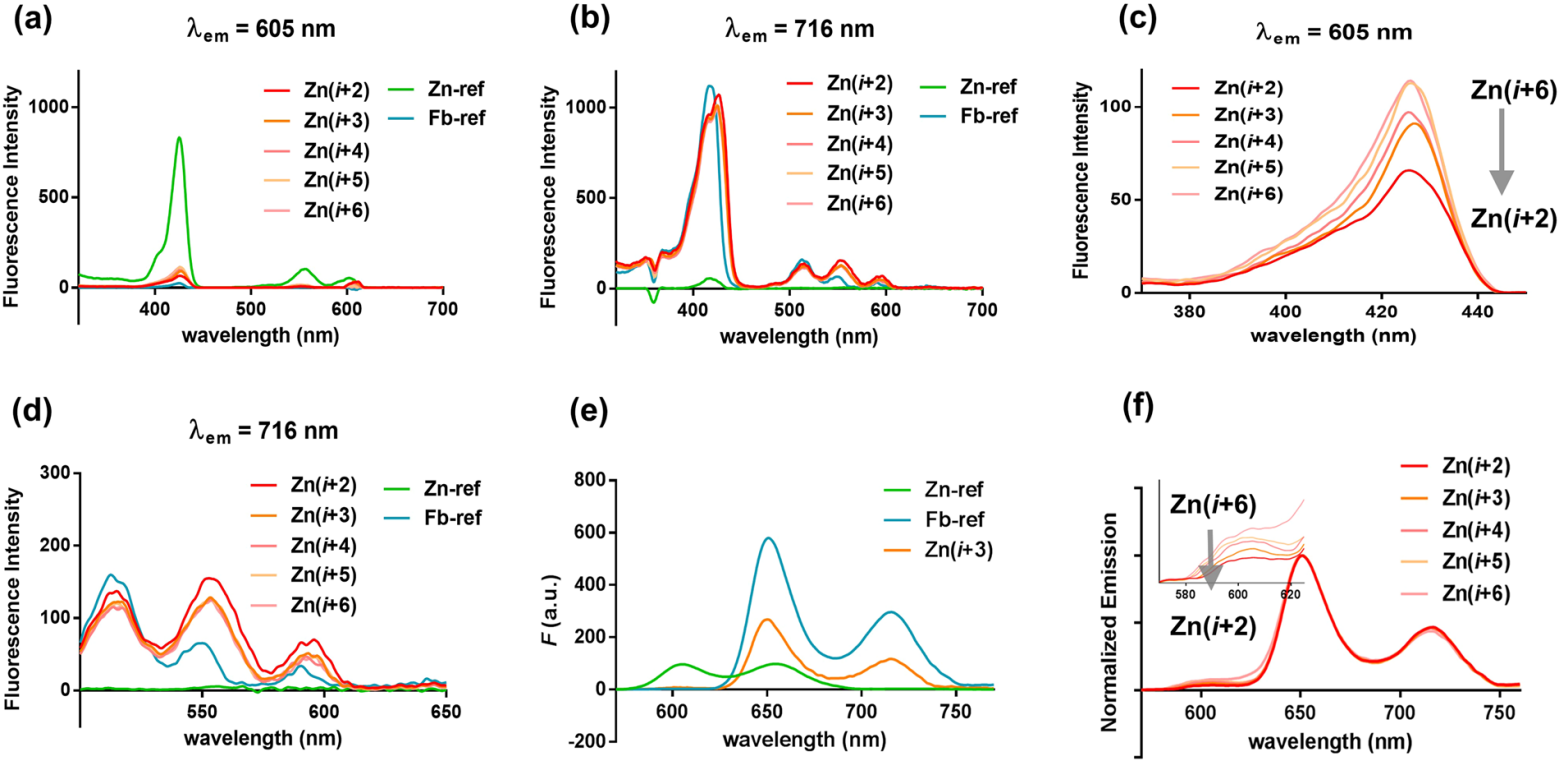
Fluorescence excitation and emission spectra of Zn PPCs: for negligible self-quenching, the samples were prepared in CH_2_Cl_2_ with concentrations of low UV-vis absorbance (0.100 ± 0.003). Excitation spectra with (**a**) ZnTPP-specific (λ_em_ = 605 nm) and (**b**) fbTPP-specific emission wavelength (λ_em_ = 716 nm). (**c**) Magnified excitation spectra of Zn PPC at the Soret region (λ_em_ = 605 nm). (**d**) Magnified excitation spectra of Zn PPC at the Q-band region (λ_em_ = 716 nm). (**e**) Fluorescence emission of **Zn(** ***i*** **+ 3)** compared to those of **Zn-ref** and **Fb-ref** at excitation wavelength of 550 nm. (**f**) Emission spectra of Zn PPCs (λ_ex_ = 550 nm): The inset shows magnified spectra around ZnTPP-specific emission (λ = 605 nm).

We monitored the fluorescence excitation and emission spectra of Zn PPCs prior to investigating the energy transfer efficiency (Fig. 6). Fluorescence excitation of Zn PPCs were recorded at two different emission wavelengths specific for donor (ZnTPP) and acceptor (fbTPP) emission (605 nm and 716 nm, respectively; Fig. 6(a)–(d)). At the emission wavelength (λ_em_) of 605 nm, **Zn-ref** showed a strong excitation spectrum with two Q-band peaks (*D*_4*h*_ symmetry of zinc porphyrin); however, the Zn PPCs showed a drastic decrease in excitation, suggesting an energy transfer from ZnTPP to fbTPP (Fig. 6(a)). Indirect information regarding the degree of energy transfer was obtained from the decreased peak intensity (which indicates an increase in the energy transfer) at 425 nm from **Zn(** ***i*** **+ 6)** to **Zn(** ***i*** **+ 2)** (Fig. 6(c)). Measurement of the excitation spectra at λ_em_ = 716 nm showed no excitation for **Zn-ref**, but typical Soret band and Q-bands for **Fb-ref** (Fig. 6(b)). Split Soret bands at 415 nm and 425 nm (from fbTPP and ZnTPP, respectively) were shown, demonstrating the dual contribution (from S_0_ to S_2_ transition) of ZnTPP and fbTPP for acceptor (fbTPP) emission (S_1_ to S_0_). The extensive spectral overlap between donor and acceptor made it difficult to identify a wavelength that solely excites the donor without contribution from direct acceptor excitation. As shown in Figs 5(b) and 6(d), donor (ZnTPP) absorbs predominantly at 550 nm, and donor excitation at this wavelength was a major contributor to acceptor emission; therefore, λ_ex_ = 550 nm was used in the following fluorescence emission and energy transfer studies^36, 37^.

With an excitation at 550 nm where the absorption of ZnTPP was more than twice that of fbTPP, all Zn PPCs showed acceptor-like emission profiles (Fig. 6(f)). A peak at 605 nm caused by donor (ZnTPP) emission was significantly diminished, indicating a highly efficient energy transfer. For each Zn PPC, the energy transfer efficiency was estimated using the equation, *E* = 1 − *F’*/*F*, where *F’* and *F* denote the fluorescence intensities of the donor in the presence and absence of the acceptor, respectively (See Supplementary page 12)^38, 39^. Absorbed energy was transferred at an efficiency of >90% in all Zn PPCs, and the energy efficiency was modulated extremely precisely over a range of 92–96%, in roughly 1% increments from **Zn(** ***i*** **+ 6)** to **Zn(** ***i*** **+ 2)** (*E*_2_ in Table 1).

**Table 1:** Energy transfer kinetics of **Zn(** ***i*** **+ 2)**-**Zn(** ***i*** **+ 6)** in toluene.

Compounds	τ_DA_ (ps)^[a]^	*k*_ET_ (s^−1^)^[b]^	*E*_1_ (%)^[c]^	*E*_2_ (%)^[d]^
Zn(*i* + 2)	64 ± 1	1.52×10^10^	97.6	96.2
Zn(*i* + 3)	67 ± 2	1.45×10^10^	97.5	94.9
Zn(*i* + 4)	68 ± 1	1.43×10^10^	97.4	93.8
Zn(*i* + 5)	87 ± 2	1.11×10^10^	96.7	93.2
Zn(*i* + 6)	97 ± 3	9.93×10^9^	96.3	91.9

[a] τ_DA_ are measured lifetimes of ZnTPP in all Zn PCCs. [b] *k*_ET_ = 1/τ_DA_ −1/τ_D_ with τ_D_ = 2.63 ns from TCSPC measurements. [c] *E*_1_ = 1 − τ_DA_/τ_D_. [d] *E*_2_ = 1 − *F’*/*F* (measured in CH_2_Cl_2_).

**Figure 7:**
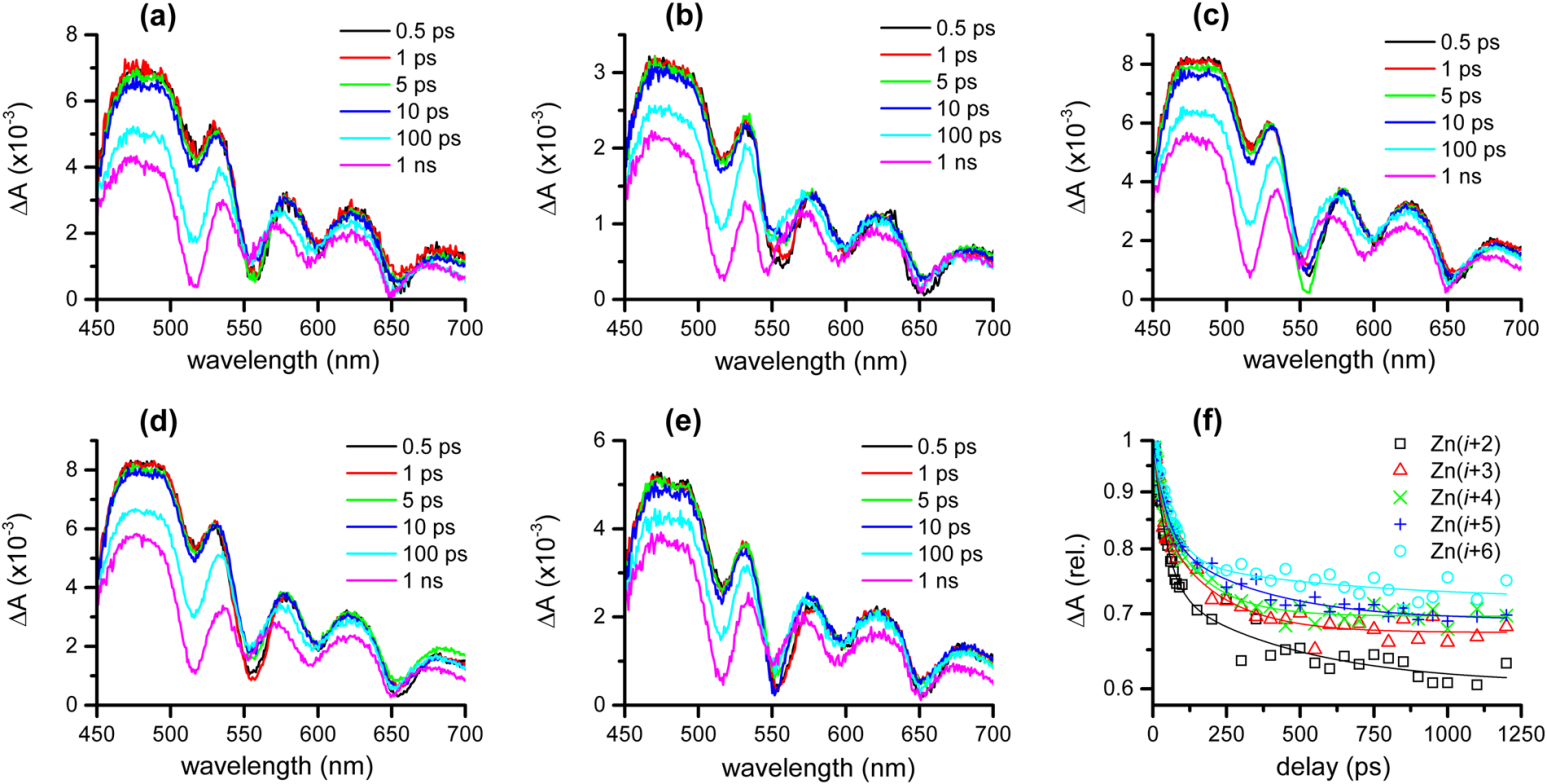
Transient absorption spectra of (**a**) **Zn(** ***i*** **+ 2)**, (**b**) **Zn(** ***i*** **+ 3)**, (**c**) **Zn(** ***i*** **+ 4)**, (**d**) **Zn(** ***i*** **+ 5)**, and (**e**) **Zn(** ***i*** **+ 6)** in toluene with the 547 nm excitation, and (**f**) kinetic traces of ESA bands at 474 nm.

**Figure 8:**
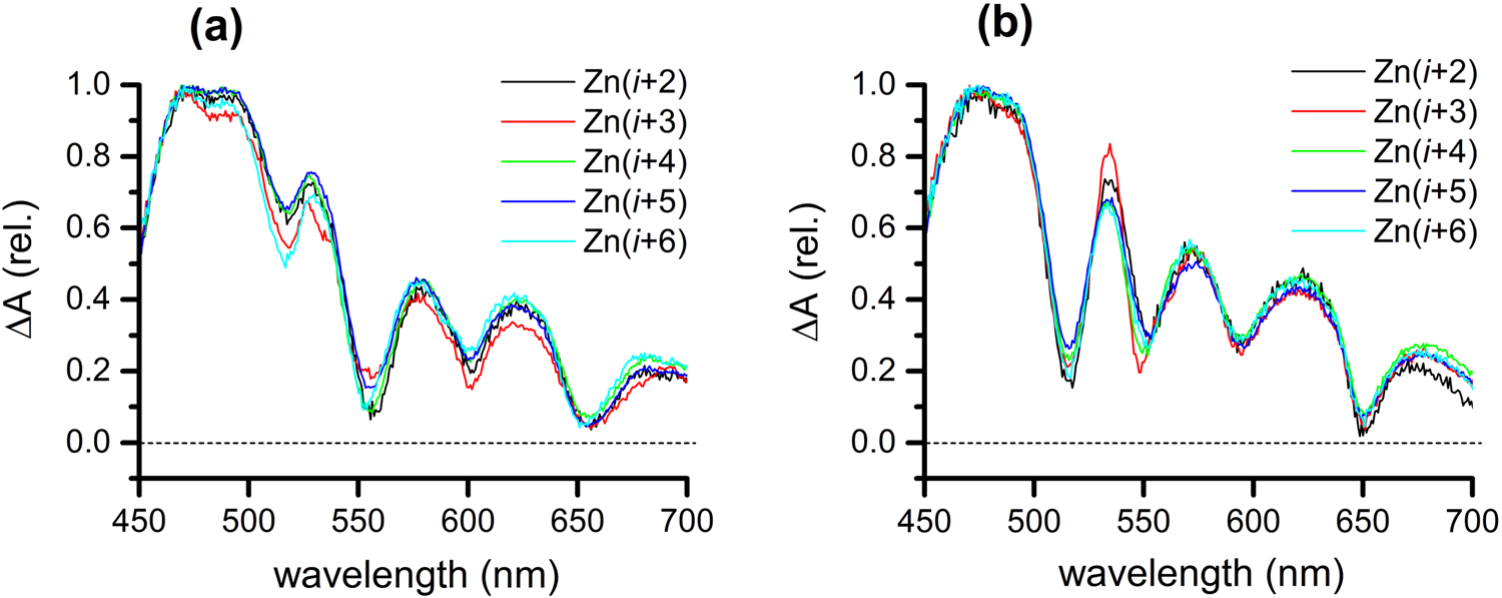
The normalized EADS for the transient absorption of **Zn(** ***i*** **+ 2)**-**Zn(** ***i*** **+ 6)** in toluene; (**a**) fast (64~97 ps) and (**b**) slow (~10 ns) components.

To measure the rate constant and efficiency of the energy transfer, femtosecond transient absorption measurements with 547 nm were performed^40, 41^. Figure 7(a)–(e) show transient absorption spectra of **Zn(** ***i*** **+ 2)**-**Zn(** ***i*** **+ 6)** in toluene, where the ground state bleaching bands at 515 and 550 nm and stimulated emission bands at 600, 650, and 720 nm (not shown) are mixed with much broader excited state absorption (ESA) bands. The kinetics of ESA bands at 474 nm, compared in Fig. 7(f), show ultrafast (65–100 ps) decay components which are considered as lifetimes of ZnTPP in Zn PPCs. The dynamics of **Zn(** ***i*** **+ 2)** was the fastest and that of **Zn(** ***i*** **+ 6)** was the slowest among Zn PPCs. Transient absorption results were further analyzed by a global analysis with a sequential model (See Supplementary page 15)^42^. Two distinct kinetic components, one with faster (64–97 ps) lifetimes and the other with much slower (~10 ns) ones, were resolved from the results of all Zn PPCs. Figure 8 shows the normalized evolution-assisted difference spectra (EADS) of two components of all Zn PPCs. The EADS of 64–97 ps lifetimes are almost identical to that of ZnTPP, and the EADS of ~10 ns lifetimes to that of fbTPP (See Supplementary Fig. S9), which confirms ultrafast energy transfer from ZnTPP to fbTPP in all Zn PPCs.

The energy transfer rate constants (*k*_ET_) are calculated from the lifetimes of ZnTPP in the Zn PPCs and in the absence of accepter fbTPP (τ_DA_ and τ_D_, respectively) using *k*_ET_ = (τ_DA_)^−1^ − (τ_D_)^−1^. The τ_D_ was measured as 2.63 ns from a separate time-correlated single photon counting (TCSPC) measurement (See Supplemenatry page 17)^43^. The energy transfer efficiency (*E*) of ZnTPP → fbTPP was also evaluated using *E* = 1 − τ_DA_/τ_D_, resulting in 97.6–96.3% for **Zn(** ***i*** **+ 2)**-**Zn(** ***i*** **+ 6)**. The energy transfer kinetics of all Zn PPCs are summarizd in Table 1.

### Molecular simulation

The energy transfer rate and efficiency increased as the donor-acceptor placement changed from (*i*, *i* + 6) to (*i*, *i* + 2). This trend and the precise modulation of energy transfer efficiency were confirmed both by static fluorescence emission and by femtosecond transient absorption spectroscopy. To gain structural insight into how the spacing of two porphyrins is influenced by peptoid sequences, we performed ~1.2 μs replica-exchange molecular dynamics (REMD) simulations^28, 44, 45^. In these simulations, we compared (*i*, *i* + 2) and (*i*, *i* + 3) PPCs with two fbTPPs attached (See Supplementary page 8–11). The simulation results show considerable conformational heterogeneity of PPCs mainly by the flexible *n*-butyl linkers conjugating the porphyrin groups. Moreover, the porphyrins are predicted to be closer together for (*i*, *i* + 2) PPC, potentially with greater excitonic coupling, than for (*i*, *i* + 3) PPC (See Supplementary Fig. S7). Based on the structural characteristics of typical poly-proline type I (PPI) peptoid helix itself^26, 29^, two porphyrins in **Zn(** ***i*** **+ 2)** and **Zn(** ***i*** **+ 3)** are expected to be slipped-cofacial and face-to-face arrangement, respectively. However, two porphyrins in **Zn(** ***i*** **+ 2)** appears to prefer face-to-face arrangement as demonstrated by the simulation result (See Supplementary Fig. S7(a)), with the shorter inter-porphyrin distance, which resulted in the greatest energy transfer rate and efficiency among all the PPCs.

## Conclusion

In this study, we developed an energy transfer system employing donor and acceptor porphyrins displayed on a peptoid helix scaffold. The PPC dyads are sufficiently well-structured, (1) maintaining close proximity between donor-acceptor porphyrins without uncontrolled porphyrin aggregations, (2) providing ultrafast energy transfer with high efficiency, and (3) modulating energy transfer efficiency depending on the relative spatial arrangement of the donor-acceptor pairs. An energy transfer system with this level of precision and tunability has not been reported so far. Therefore, we believe that this system could be a fundamental research tool to understand various energy transfer events, including natural photosynthetic light harvesting. Employing nature’s design principle, sophisticated pigment-helix conjugates that can mimic natural light-harvesting molecular apparatus are currently being synthesized.

## Electronic supplementary material

Supplementary Information
