# Supplementary material for: Precisely tuneable energy transfer system using peptoid helix-based molecular scaffold
Source: Sci Rep. 2017 Jul 6;7:4786. doi: 10.1038/s41598-017-04727-0 (PMC5500559; doi:10.1038/s41598-017-04727-0)
Supplement: Supplementary file 1 — Supplementary Information [file 41598_2017_4727_MOESM1_ESM.pdf]

Supplementary Information

Precisely tunable energy transfer system using peptoid helix-based molecular scaffold

Boyeong Kang,[a] Woojin Yang,[a] Sebok Lee,[a] Sudipto Mukherjee,[b] Jonathan Forstater,[b] Hanna Kim,[a] Byoungsook Goh,[a] Tae-Young Kim,[a,c] Vincent A. Voelz,*[b] Yoonsoo Pang,*[a] Jiwon Seo*[a]

[a] Department of Chemistry, School of Physics and Chemistry, [c] School of Earth Sciences and Engineering, Gwangju Institute of Science and Technology, Gwangju 61005, Republic of Korea
[b] Department of Chemistry, Temple University, Philadelphia, PA 19122, USA

Email: jseo@gist.ac.kr (J. Seo), ypang@gist.ac.kr (Y. Pang), voelz@temple.edu (V. A. Voelz)

Table of contents

1. Synthesis 2-3
Figure S1. Synthetic routes for fbTPP-COOH, fbTPP-NHS, ZnTPP-COOH, and ZnTPP-NHS.

2. HPLC data 4-6
Figure S2. HPLC chromatogram of Fb(*i*+*n*), Pep(*i*+*n*) and Fb-ref with UV detection at 220 nm.
Figure S3. HPLC chromatogram of Zn(*i*+*n*) and Zn-ref with UV detection at 220 nm.
Figure S4. HPLC chromatogram of Fb-ref and Zn-ref.

3. ESI-MS data 7
Table S1. ESI-MS data of PPCs.

4. Molecular simulations of porphyrin-peptoid conjugates (PPCs) 8-11
Figure S5. Sampling convergence of REMD simulations.
Figure S6. Maintenance of helical folds for (*i*, *i*+2) and (*i*, *i*+3) PPCs.
Figure S7. Simulated inter-porphyrin distances and angles for (*i*, *i*+2) and (*i*, *i*+3) PPCs.

5. Calculation of energy transfer efficiency 12
Table S2. Emission intensity at 605 nm (F_{605}) and estimated energy transfer efficiencies of Zn PPCs.

6. Time-resolved fluorescence of fbTPP and ZnTPP 13
Figure S8. Emission kinetics of (a) fbTPP and (b) ZnTPP in toluene by TCSPC measurements.

7. Transient absorption spectroscopy 14
Figure S9. Transient absorption spectra of (a) ZnTPP and (b) fbTPP in toluene with the 547 nm excitation, and the EADS of (c) ZnTPP and (d) fbTPP by the global analysis.

8. Global analysis 15
Figure S10. The global analysis results for the transient absorption of Zn(*i*+2)-Zn(*i*+6) in toluene.

9. References 16-17

1. Synthesis

Synthesis of porphyrins

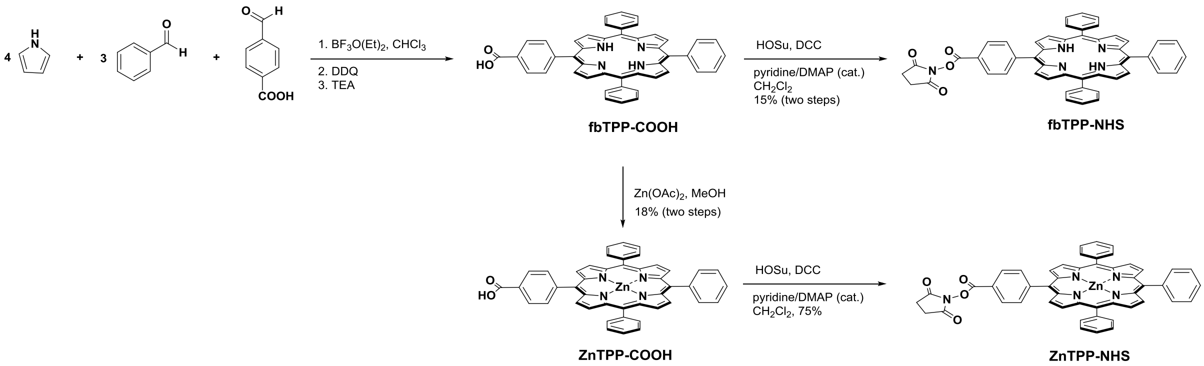

Figure S1. Synthetic routes for **fbTPP-COOH**, **fbTPP-NHS**, **ZnTPP-COOH**, and **ZnTPP-NHS**.

Initially, synthesis of **fbTPP-COOH** (5-(4-carboxyphenyl)-10,15,20-triphenylporphyrin) was attempted using Adler's protocol.[1] However, as Fungo et al. noted,[2] the reaction work up was cumbersome due to the difficulty of removing propionic acid and the insoluble tar side products, which resulted in poor yield. Hence, we employed Lindsey's method,[3] which we found to be more efficient. A dried round bottom flask was charged with 4-formylbenzoic acid (1 g, 6.66 mmol), benzaldehyde (2.1 g, 19.98 mmol, 2.03 ml) and pyrrole (1.79 g, 26.64 mmol, 1.85 ml), and then $CHCl_3$ (280 ml, with 0.75% ethanol, purchased from Sigma Aldrich) was added. To this mixture was added $BF_3{\cdot}OEt_2$ (0.94 g, 6.66 mmol, 0.818 ml, redistilled and ≥ 46.5% BF_3 basis, purchased from Sigma Aldrich) dropwise. The flask was sealed with rubber septum, and the mixture was stirred for 1 h at room temperature under N_2 atmosphere. After 1 h, 2,3-dichloro-5,6-dicyano-1,4-benzoquinone (DDQ, 4.54 g, 19.98 mmol) was added, and the oxidation reaction was kept for 1 h. The reaction was quenched by slow addition of triethylamine (1.01 g, 9.99 mmol, 1.39 ml), and the reaction mixture was stirred for another 30 minutes. The mixture was concentrated under reduced pressure, and the resulting dark purple residue was purified by short silica gel plug to afford **fbTPP-COOH** (5-(4-carboxyphenyl)-10,15,20-triphenylporphyrin) (DCM: MeOH = 20 : 1, R_f = 0.3). The mono-carboxylate form of fbTPP was difficult to be obtained in a high purity by single silica gel chromatography. Instead of repeated purifications, we moved on to the next step to obtain an NHS ester, which was readily purified by silica gel chromatography.

To a dry round bottom flask was added *N*-hydroxysuccinimide (0.57 g, 0.49 mmol), *N,N'*-dicyclohexylcarbodiimide (0.10 g, 0.49 mmol) and **fbTPP-COOH** (approximately 130 mg, 0.20 mmol), followed by DCM (6 ml). Then, pyridine (39 mg, 0.49 mmol, 0.040 ml) and a catalytic amount of 4-dimethylaminopyridine were added into the flask. The mixture was stirred overnight at room temperature. The reaction mixture was then evaporated by rotary evaporator, and the residue was purified by flash column chromatography (DCM 100%, R_f = 0.35). **fbTPP-NHS** ester was obtained as purple film with roughly 15% yield over 2 steps. Spectral data matched with the previously reported.[4]

ZnTPP-COOH and **ZnTPP-NHS** were synthesized following the procedure reported by Matsumoto et al.[5] In brief, $Zn(OAc)_2$ (3.10 g, 22.3 mmol) in MeOH (75 mL) was added to **fbTPP-COOH** (1.47 g, 2.23 mmol) in DCM (35 ml) and stirred overnight at room temperature. The mixture was concentrated, dissolved in DCM, and

washed with water to remove excess zinc acetate. Collected dichloromethane layer was evaporated by rotary evaporator, and the residue was purified by flash column chromatography (DCM : MeOH = 20 : 1, R_f = 0.35). **ZnTPP-COOH** was obtained in 18% yield over two steps. For the conversion of **ZnTPP-COOH** to **ZnTPP-NHS**, the above procedure for **fbTPP-NHS** was used (75% yield, DCM 100%, R_f = 0.17).

Synthesis of porphyrin-peptoid conjugates displaying one Zn porphyrin and one free base porphyrin (Zn PPCs)

Porphyrin conjugation

Initially, the conjugation of Zn porphyrin was attempted with compound **2** using **ZnTPP-NHS**. ZnTPP conjugation was performed under basic conditions; however, analytical HPLC or ESI-MS showed no product. The data indicated that **ZnTPP-NHS** and even compound **2** were decomposed under the reaction conditions (data not shown). Alternatively, the conjugation of Zn porphyrin with **ZnTPP-COOH** was attempted, without using the NHS ester (Figure S1). The coupling agents HATU, HCTU, and COMU were tested with bases with different pK_a values that are generally used in peptide coupling reactions: DIEA (*N*,*N*-diisopropylethylamine; pK_a = 10.7) and NMM (*N*-methylmorpholine; pK_a = 7.4). Unfortunately, the yields under these reaction conditions were poor; it is likely that the interactions between Zn porphyrin and coupling reagent compete with the coupling reaction, resulting in reduced efficiency.[6] At this point, we decided to avoid ZnTPP coupling to compound **2**; instead, metalation of compound **2**, followed by fbTPP conjugation appeared to be a feasible alternative.

Solid phase extraction (SPE) purification

Demetallation of Zn readily occurs under acidic conditions. Since we used 0.1% TFA containing buffer as HPLC mobile phases, collected fractions after preparative HPLC contained small amount of TFA. Routine practice was to lyophilize the fractions containing desired product; however, we observed release of Zn^{2+} ions from porphyrin during the lyophilization, lowering purity of the final product. Therefore, SPE (solid phase extraction) was used to remove the small amount of TFA before concentrating the HPLC fractions. SPE cartridges were purchased from Waters (Oasis HLB 6 cc, 500 mg). HPLC grade ACN and anhydrous DCM (> 99.8%) were used. To control the flow rate, vacuum manifold (purchased from Sigma Aldrich) was used. On the cartridge, conditioning was performed firstly using DCM (4 ml) and ACN (10 ml) under 5 mmHg. Flow rate was kept approximately 2 drops per second. After loading 1 ml of collected fraction from preparative HPLC, washing was carried out using ACN (10 ml) by gravity. For elution, DCM (1 ml) mixed with ACN (2 ml) was firstly used followed by DCM (1.5 ml) under 5 mmHg with speed of 2 drops per second. During these steps, product did not elute yet from the column. Right after, DCM elution (1 ml) was consecutively performed for two times. Finally, Zn PPC dissolved in DCM without TFA was obtained. Collected elute solution from each SPE run was concentrated, lyophilized and stored at -80 °C.

2. HPLC data

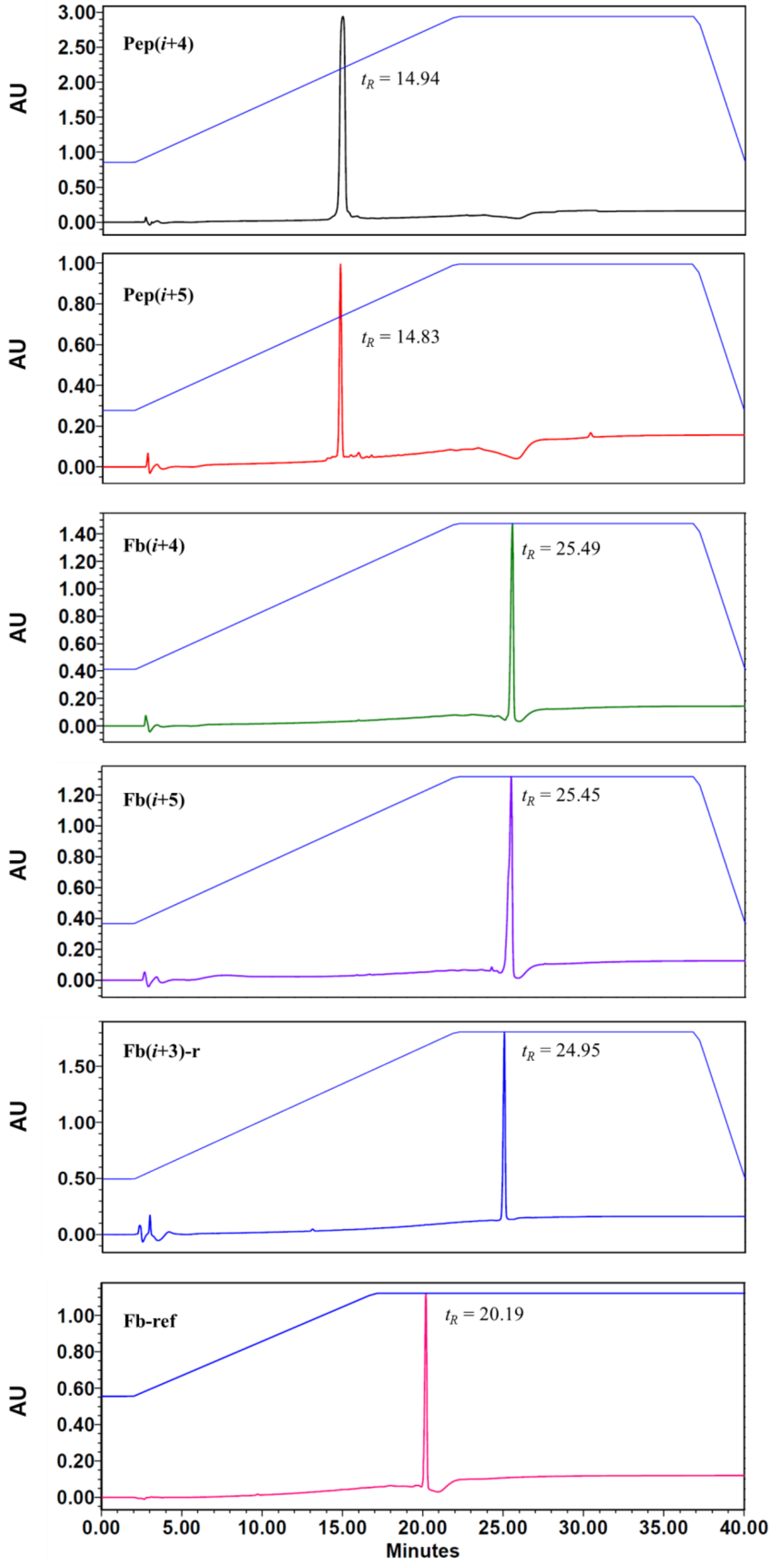

Figure S2. HPLC chromatogram of **Fb(*i*+*n*)**, their reference peptoids (**Pep(*i*+*n*)**) and **Fb-ref** with UV detection at 220 nm.

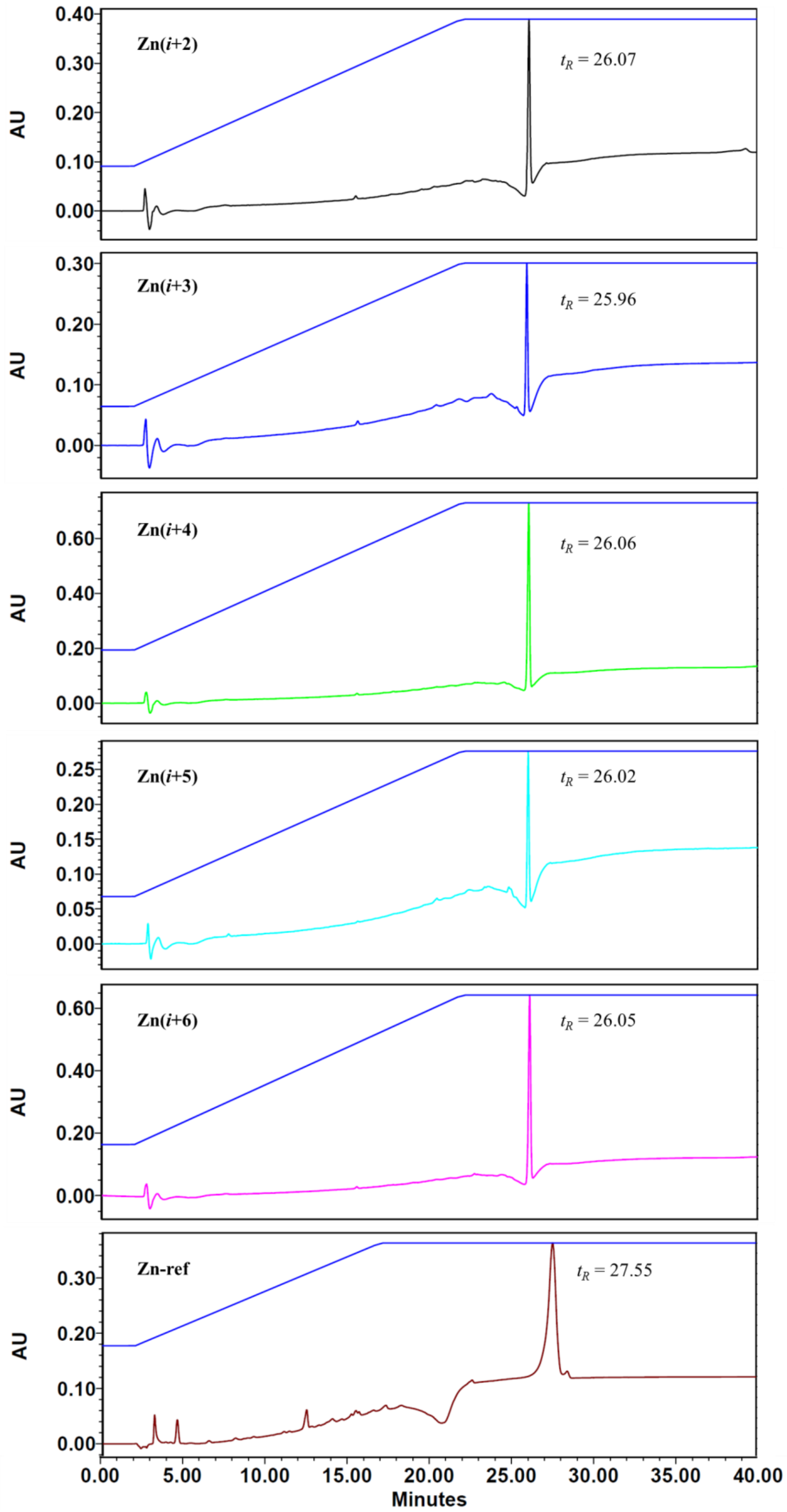

Figure S3. HPLC chromatogram of **Zn(*i*+*n*)** and **Zn-ref** with UV detection at 220 nm.

Interestingly, evidence of self-assembly of **Zn-ref** was observed during HPLC analysis. Unusually broadened bell-shape peak was shown at about 2 times longer retention time compared to **Fb-ref**. The peak broadening can be attributed to the heterogeneous assembled species of **Zn-ref**. At an elevated temperature of 60 °C, the peak at 44.3 minutes moved to 27.5 minutes, and the peak shape was sharpened. Compared to **Fb-ref**, zinc atom is coordinated by acetonitrile molecules resulting in the less hydration and longer retention of **Zn-ref** in reversed-phase HPLC.

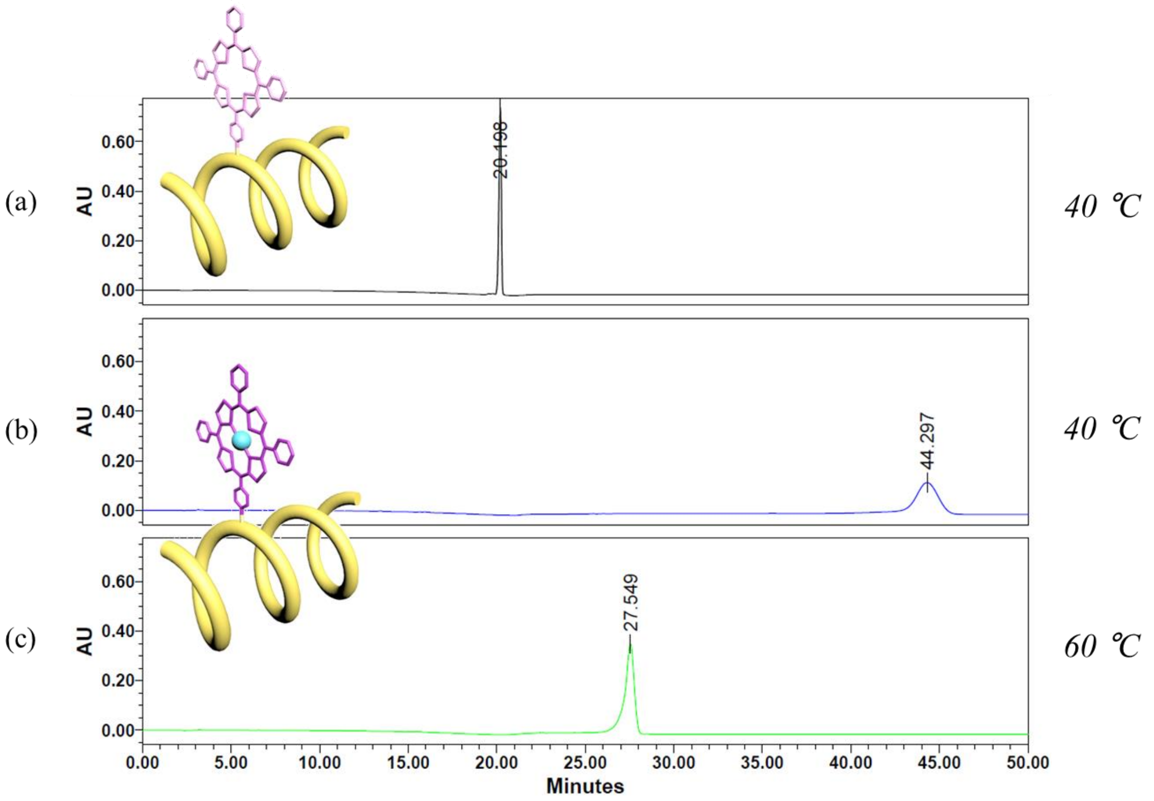

Figure S4. HPLC chromatogram of **Fb-ref** and **Zn-ref** monitored at 410 nm. Retention time was indicated on each peak. (a) **Fb-ref** at 40 °C. (b) **Zn-ref** at 40 °C. (c) **Zn-ref** at 60 °C.

3. ESI-MS data

Compounds	Mass calculated	Mass observed[a]
**Pep(*i*+4)**	1926.07	1927.07 (H^+), 1949.05 (Na^+)
**Pep(*i*+5)**	1926.07	1927.07 (H^+), 1949.05 (Na^+)
**Fb(*i*+4)**[b]	3206.52	1604.27 ($2H^+$), 1627.25 ($2Na^+$)
**Fb(*i*+5)**[b]	3206.52	1604.27 ($2H^+$), 1627.25 ($2Na^+$)
**Fb(*i*+3)-r**	2723.27	2725.27 (H^+), 1362.64 ($2H^+$)
**Fb-ref**	2116.03	2117.05 (H+), 1059.02 (2H+)
**Zn(*i*+2)**	2785.18	1393.59 ($2H^+$), 1415.58 ($2Na^+$)
**Zn(*i*+3)**	2785.18	1393.60 ($2H^+$), 1415.58 ($2Na^+$)
**Zn(*i*+4)**[b]	3268.43	1635.26 ($2H^+$), 1657.24 ($2Na^+$)
**Zn(*i*+5)**[b]	3268.43	1635.26 ($2H^+$), 1657.24 ($2Na^+$)
**Zn(*i*+6)**[b]	3268.43	1635.26 ($2H^+$), 1657.24 ($2Na^+$)
**Zn-ref**	2177.94	2202.95 (Na^+), 1112.96 ($2Na^+$)

Table S1. ESI-MS data of PPCs. [a] Observed in ESI-MS. [b] Singly charged species were not observed because of the mass range of instrument (up to *m/z* 3000). Observed masses are not fragments but doubly charged ($2H^+$) peaks.

4. Molecular simulations of porphyrin-peptoid conjugates (PPCs)

To gain insight into how peptoid conformational properties are affected by the spacing of porphyrin-conjugated residues, we performed ~1.2 μs replica-exchange molecular dynamics (REMD) simulations of (*i*, *i*+2) and (*i*, *i*+3) PPCs with two fbTPPs attached (or **Fb(*i*+2)** and **Fb(*i*+3)**, respectively)7, using methods similar to previous approaches.[8,9,10] In these simulations, 20 or 24 temperature replicas ranging from 300 K to 800 K, with nearest-temperature exchange attempts every 10 ps, were used to efficiently sample peptoid conformations separated by large *cis*/*trans* amide barriers trajectory. As each replica performs a random walk in temperature, omega-angles of all peptoid residues decorrelate over timescales ranging from 20 to 400 ns, indicating converged sampling.

We define a peptoid residue to be helical if it has a *cis*-amide and a negative backbone phi angle. Per-residue *cis*-amide populations and helix populations show that the helical fold is maintained for both **Fb(*i*+2)** (56% helix) and **Fb(*i*+3)** (41% helix), with helicity tracking closely with the *cis*-amide population (Figure S6). To explore how the conformational landscapes of **Fb(*i*+2)** and **Fb(*i*+3)** might help tune exciton coupling and energy transfer efficiency, we projected the 300 K simulation data to two key structural observables: the inter-porphyrin distance, and the angle (θ) made between normal vectors of the planar porphyrin rings.

The results show considerable conformational heterogeneity, made possible by the many available peptoid backbone configurations, as well as the flexible *n*-butyl linkers conjugating the porphyrin groups. Despite this flexibility, three specific conformational basins can be identified: one corresponding to porphyrin groups stacked in close proximity (< 0.9 nm); another with porphyrin groups farther apart (~1.2 nm) staggered at nearly right angles; and a third basin with the porphyrins ~1.4 nm apart in a more planar orientation (Figure S7). The simulations predict that the first and second such basins are more populated for **Fb(*i*+2)**, while the second and third basins are more populated for **Fb(*i*+3)**. This result means that the porphyrins are predicted to be closer together for **Fb(*i*+2)**, potentially with greater excitonic coupling. This picture is consistent with expectations for flexible polymers, as **Fb(*i*+3)** should have a greater entropic penalty for bringing the porphyrin groups in close proximity compared to **Fb(*i*+2)**. For **Fb(*i*+3)**, the greater spacing between the porphyrin groups favors conformations with large inter-porphyrin distances, including a population peptoid scaffold is sandwiched between nearly planar porphyrin groups (Figure S7(f)). The low helicity of such states likely contributes to an overall decrease in helicity for **Fb(*i*+3)** versus **Fb(*i*+2)** observed experimentally.

System preparation.

Molecular topologies for molecules **Fb(*i*+2)** and **Fb(*i*+3)** were constructed using the General Amber Force Field (GAFF)[11] with partial changes from AM1-BCC.[12] The GAFF+φ torsion correction was applied to all peptoid residues, as described in Mukherjee et al.[10]

MD simulation. (1.2 μs × 24 (or 20) temperatures replicas from 300 K to 800 K)

The OBC Generalized Born implicit solvation model was used in all simulations.[13] Swaps between adjacent replicas were attempted every 10 ps, with acceptance ratios ranging from 0.45 to 0.51. Simulations were performed with GROMACS 4.6 dynamics package. Stochastic integration (Langevin dynamics) was used with a

2 fs time step and water-like viscosity. Trajectory snapshots and energies were written every 10 ps. The initial starting structure of each replica was a right-handed helical conformation with an all-*cis* amide backbone.

Sampling convergence was assessed using autocorrelation analysis (Figure S5). The MDTraj package[14] was used calculate structural observables (*cis*-amide populations, helix populations, inter-porphyrin distances and angles) from samples after 500 ns at 300 K. Normal vectors for each porphyrin ring were calculated as the mean cross-product of sequential nitrogen-nitrogen distance vectors around the ring. As an additional test of the robustness of the results, we also performed simulations in which the GAFF+φ torsion correction was applied to all residues, including Nlys(fbTPP). Similar results were obtained in all cases (Figure S6(B)) with the main difference being a slight increase in overall helicity for both **Fb(*i*+2)** (65% helix) and **Fb(*i*+3)** (62% helix).

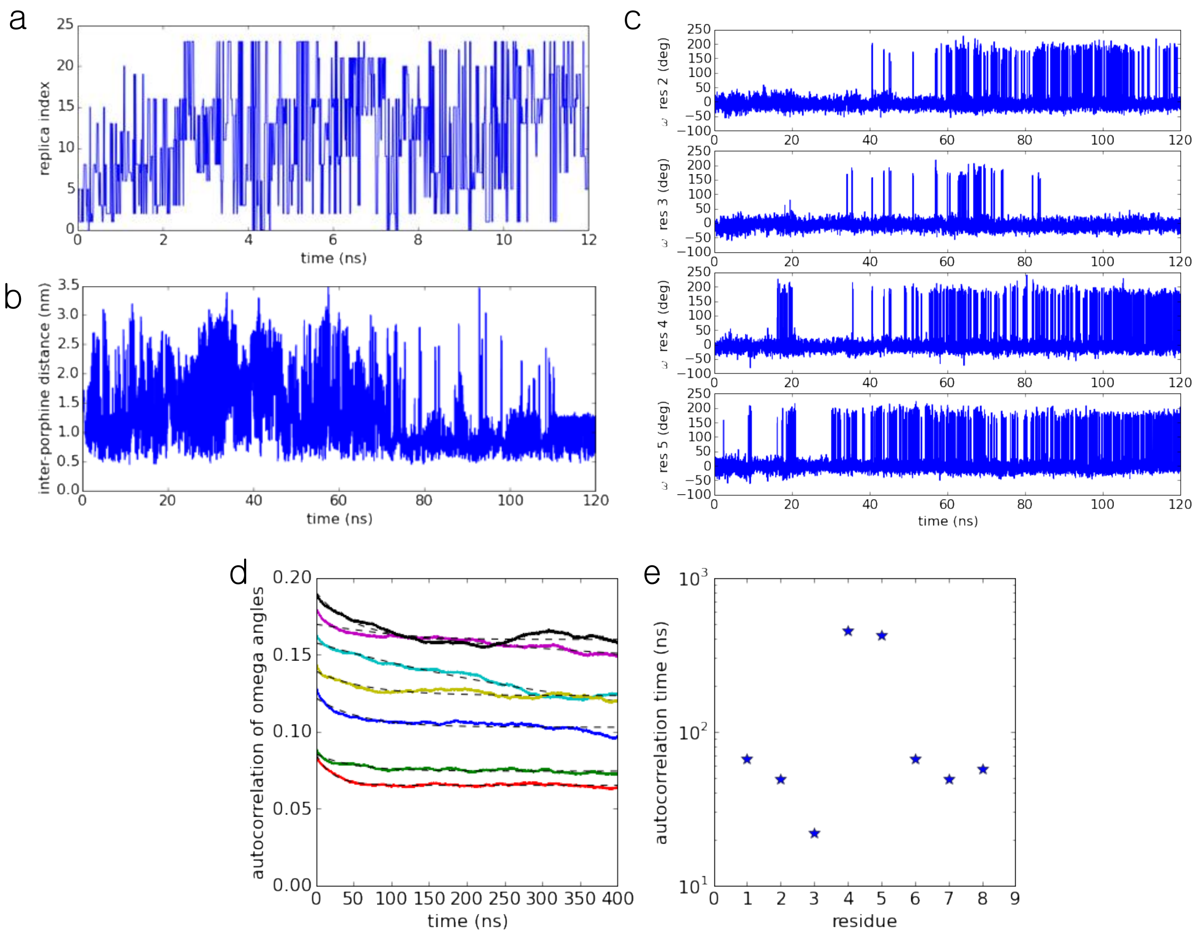

Figure S5. Sampling convergence of REMD simulations. (a) Random walks in temperature space traverse the entire temperature range in nanoseconds in a representative REMD trajectory. (b) Inter-porphine distances sample a wide range of values over nanoseconds as each replica samples different temperatures. (c) Representative traces of backbone amide ω angles over time show slow barrier crossings for *cis*/*trans* amide isomerization. (d) Autocorrelation functions $\langle\chi(\omega(t))\chi(\omega(t+\tau))\rangle$, where $\chi(\omega) = 0$ for *cis* amides and $\chi(\omega)=1$ for *trans* amides, fit to single exponential functions $a_0 + a_1 \exp(-t/\tau_c)$. (e) Correlation times τ ranging from ~20 - 400 ns for **Fb(*i*+2)**. Since our simulations are ~3x longer than this, we can be reasonably sure that ω-angle populations are well sampled.

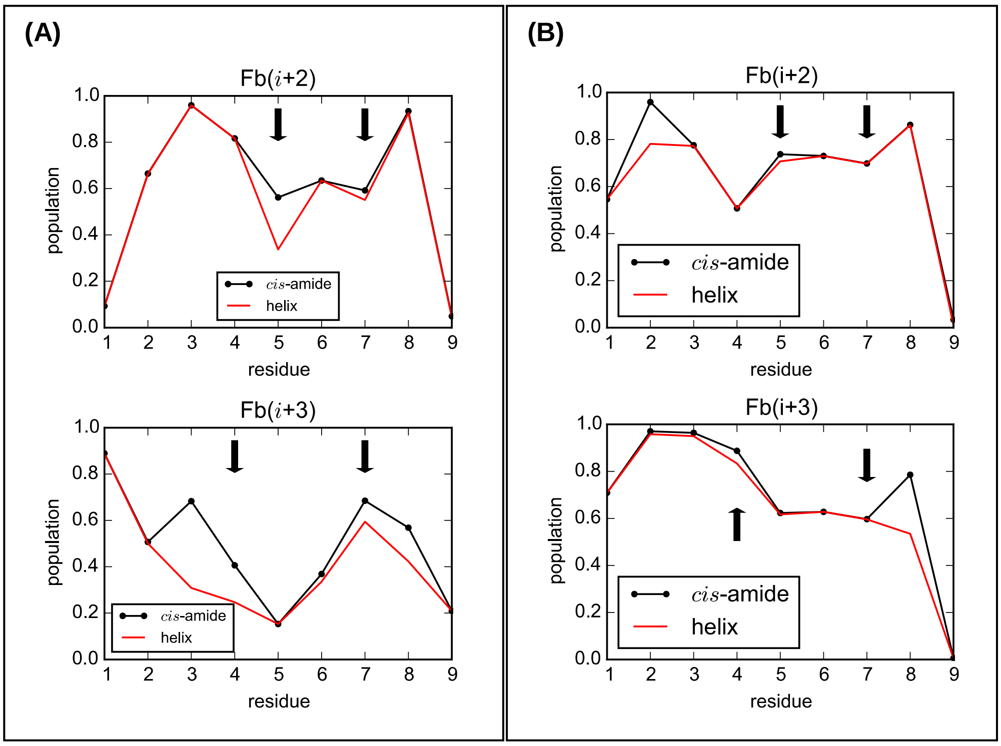

Figure S6. Maintenance of helical folds for **Fb(*i*+2)** and **Fb(*i*+3)** for simulations before **(A)** or after **(B)** the GAFF+φ torsion correction was applied to all residues. Shown are cis-amide (black) and helix (red) populations for each residue. Arrows mark the location of the porphyrin-conjugated *N*lys(fbTPP) residues. Residue numbers are given from *N*- to *C*-termini.

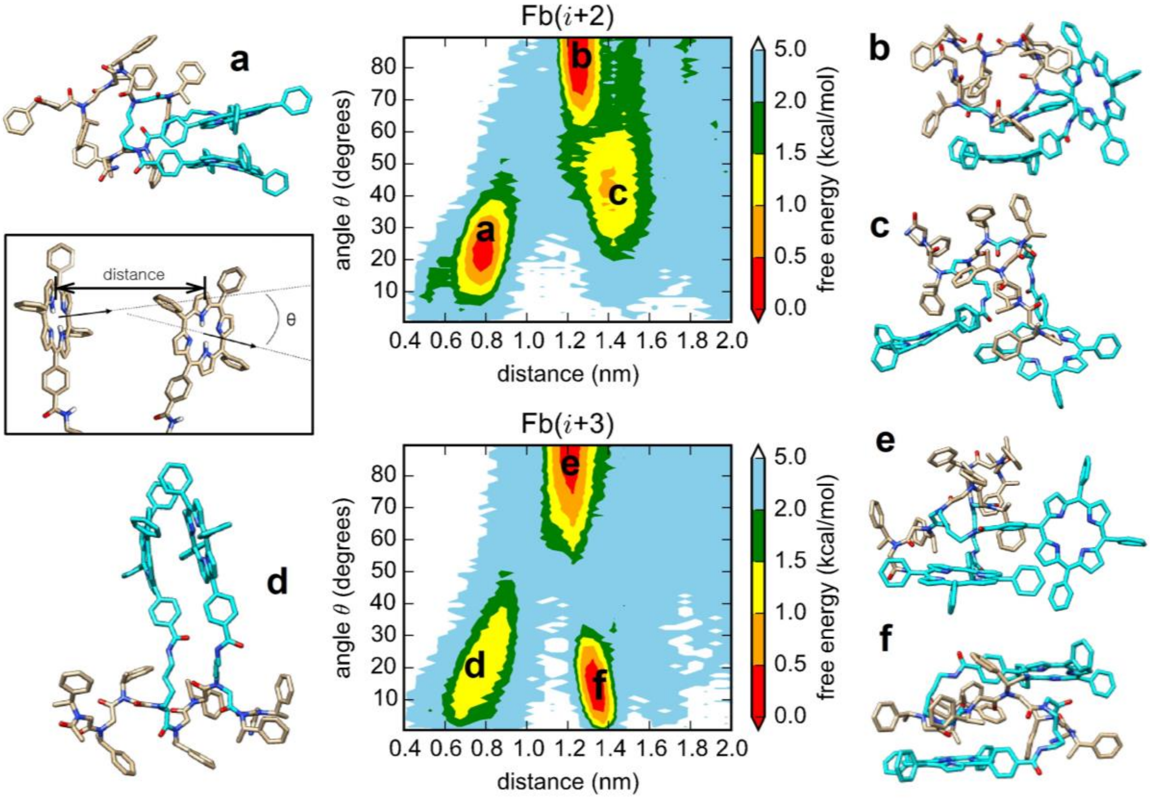

Figure S7. Simulated inter-porphyrin distances and angles for **Fb(*i*+2)** and **Fb(*i*+3)**. Distances are calculated from the centers of each porphyrin ring, and angles from the orientation of vectors normal to each ring plane. Three main conformational basins can be identified on the free energy landscape for **Fb(*i*+2)** (a-c) and **Fb(*i*+3)** (d-f), each shown with a representative conformation from the simulations. Among the predicted conformations, only (a) and (d) of **Fb(*i*+2)** and **Fb(*i*+3)**, respectively, show maintenance of helical peptoid conformation, which agrees with our previous experimental data (e.g., UV-vis and circular dichroism spectroscopy).[7]

5. Calculation of energy transfer efficiency

To estimate the energy transfer efficiency of each Zn PPC, the ratio of emission intensity (i.e., F_{605}) between **Zn-ref** and **Zn(*i*+*n*)** was used (Table S2), and FRET efficiency was calculated by $E = 1 - F'/F$, where F' and F are fluorescence intensity of a donor in the presence and absence of an acceptor, respectively.

Compounds	F_{605}	E (%)
**Zn-ref**	0.99	
**Zn(*i*+2)**	0.037	96.2
**Zn(*i*+3)**	0.050	94.9
**Zn(*i*+4)**	0.061	93.8
**Zn(*i*+5)**	0.067	93.2
**Zn(*i*+6)**	0.080	91.9

Table S2. Emission intensity at 605 nm (F_{605}) and estimated energy transfer efficiencies of Zn PPCs. The samples were prepared in CH_2Cl_2 with concentrations of low UV-vis absorbance (0.100 ± 0.003).

6. Time-resolved fluorescence of fbTPP and ZnTPP

To measure the lifetimes of fbTPP and ZnTPP in toluene, time-correlated single photon counting (TCSPC) measurements with 405 nm excitation were performed. A home-built TCSPC setup based on a TCSPC module (PicoHarp 300, PicoQuant) and a photomultiplier tube (PMA 192, PicoQuant) was used.[15] Absorbance of all samples was kept below 0.1 and the pulse energy of 0.5 pJ at 10 MHz was used. The time-resolved fluorescence kinetics of fbTPP and ZnTPP probed at the selected wavelengths near the maximum of the fluorescence spectra of fbTPP (650 and 710 nm) and ZnTPP (600 and 650 nm) in toluene were shown in Figure S8.

The emission kinetics of fbTPP and ZnTPP were fit by a convoluted model (shown in equation S1) between the Gaussian instrumental response function and an exponential decay function,

$$\Delta A(t) = A_0 + A_1 \exp\left[\frac{\omega^2}{2\tau^2} - \frac{t-t_0}{\tau}\right]\left[1 - erf\left(\frac{\omega^2 - \tau(t-t_0)}{\sqrt{2}\omega\tau}\right)\right] \quad \text{(S1)}$$

where A_0 is a constant, A_1 is an amplitude, t_0 is the position of time zero, and ω is related to the pulsewidth of instrumental Gaussian function (pulsewidth = 2.325 × ω), and τ is the lifetime of an exponential decay. The emission lifetimes of fbTPP and ZnTPP were determined as 12.2 ns and 2.63 ns, respectively.

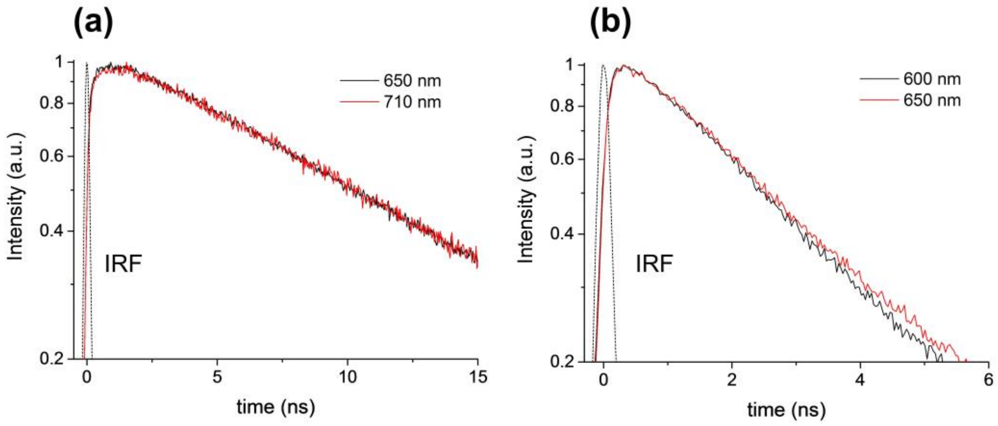

Figure S8. Emission kinetics of (a) fbTPP (probed at 650 and 710 nm) and (b) ZnTPP (probed at 600 and 650 nm) in toluene by TCSPC measurements.

7. Transient absorption spectroscopy

A femtosecond transient absorption spectroscopy setup based on a Ti:sapphire laser system (LIBRA-USP-HE, Coherent, Inc.; <50 fs, 1 kHz repetition) was used to measure time-resolved spectra of fbTPP, ZnTPP, and Zn PPCs.[16,17] The 547 nm pump pulses with ~35 nm FWHM were generated from a home-built non-collinear optically parametric amplifier and compressed in a prism pair compressor. A broadband probe pulses (450-1000 nm) generated from the supercontinuum generation in a sapphire window were measured in a fiber-based spectrometer (QE65Pro, Ocean Optics).[16,17]

Figure S9 shows transient absorption spectra of ZnTPP and fbTPP in toluene measured with 547 nm excitation, and the evolution-associated difference spectra (EADS) of ZnTPP and fbTPP as the result of the global analysis. The transient absorption spectra of ZnTPP were composed of the ground state bleaching (GSB) band at 550 nm and stimulated emission (SE) bands at 600 and 650 nm, which were mixed with the very broad excited state absorption (ESA) band of which maximum was at 475 nm. The transient absorption spectra of fbTPP were also composed of the GSB band at 520 nm and stimulated emission (SE) bands at 550,590, 650, and 725 nm, which were mixed with the very broad ESA band of which maximum was at ~500 nm.

The transient absorption kinetics of ZnTPP and fbTPP in toluene were fit by the global analysis, where an exponential decay component of 2.6 ns and ~10 ns was observed for ZnTPP and fbTPP, respectively.

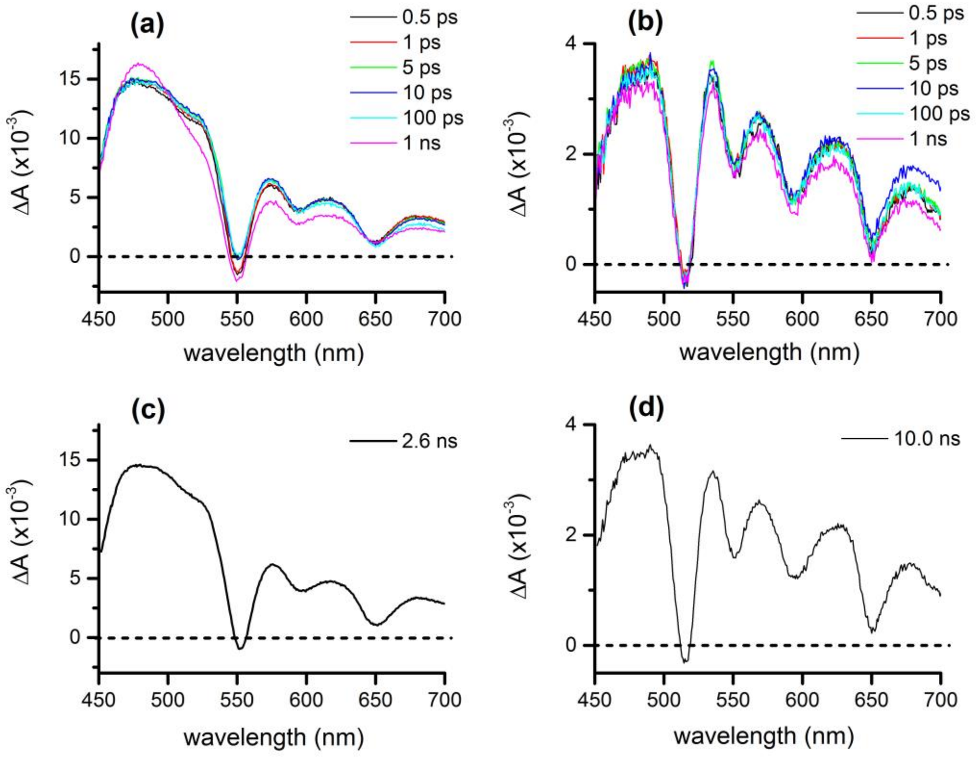

Figure S9. Transient absorption spectra of (a) ZnTPP and (b) fbTPP in toluene with the 547 nm excitation, and the EADS of (c) ZnTPP and (d) fbTPP by the global analysis.

8. Global analysis

The kinetics for the excited-state dynamics and ultrafast energy transfers of transient absorption signals were analyzed by the global fit offered in the Glotaran software.[18,19] The time-resolved spectra, $\Psi(t,\lambda)$, are analyzed as a superposition of the specific kinetic components as shown in equation S2,

$$\Psi(t,\lambda) = \sum_{i=1}^{n} c_i(t)\varepsilon_i(\lambda) \tag{S2}$$

where $c_i(t)$ and $\varepsilon_i(\lambda)$ represent the time evolution of concentration and the spectrum of the kinetic component associated with the rate constant, k_i, respectively. The spectrum of each kinetic component, $\varepsilon_i(\lambda)$, is called the decay-associated (difference) spectra (DAS or DADS for a parallel decay model), the evolution-associated (difference) spectra (EAS or EADS for a sequential decay model), or the species-associated (difference) spectra (SAS or SADS for a branching decay model).

The ultrafast energy transfers in all Zn PPCs observed in the transient absorption measurements were analyzed by the global fit with the sequential decay model; a fast (64-97 ps in all Zn PPCs) component representing the energy transfer from ZnTPP to fbTPP and a slower (~10 ns in all Zn PPCs) component representing the lifetime of fbTPP. The results of global analysis with the sequential model for the transient absorption spectra of all Zn PPCs were shown in Figure S10, where all transient absorption kinetics were well represented by the fit model.

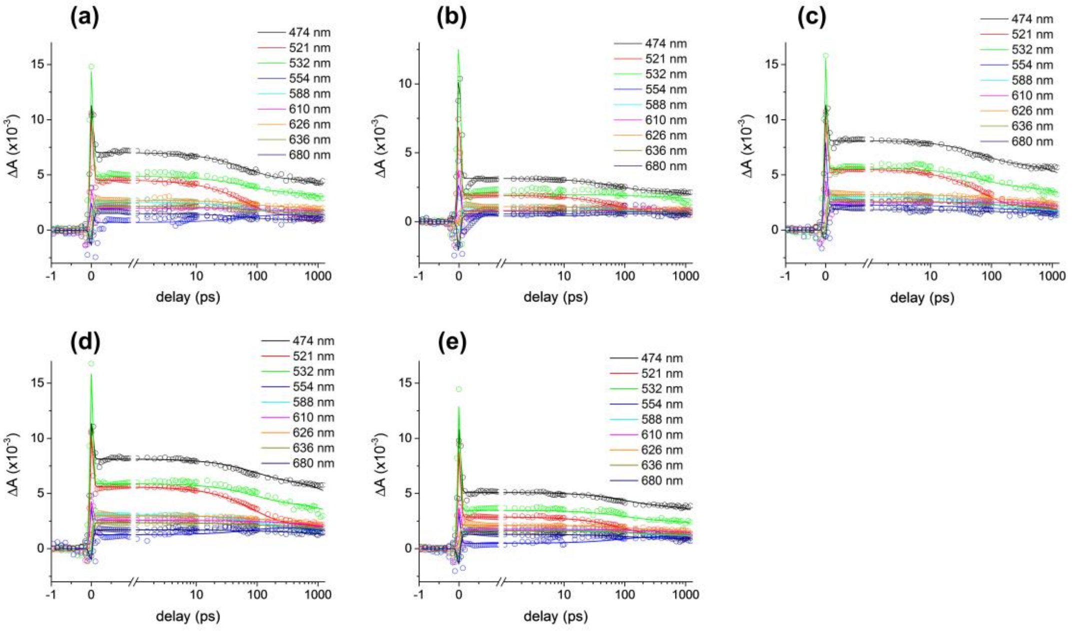

Figure S10. The global analysis results for the transient absorption of **Zn(*i*+2)-Zn(*i*+6)** in toluene; observed values (open circles) and corresponding fit lines were shown for several probe wavelengths.

9. References

1. Adler, A. D. *et al.* A simplified synthesis for meso-tetraphenylporphine. *J. Org. Chem.* **32**, 476 (1967).

2. Fungo, F., Otero, L. A., Sereno, L. Silber, J. J. & Durantini, E. N. Synthesis of porphyrin dyads with potential use in solar energy conversion. *J. Mater. Chem.* **10**, 645-650 (2000).

3. Lindsey, J. S., Schreiman, I. C., Hsu, H. C., Kearney P. C. & Marguerettaz, A. M. Rothemund and Adler-Longo reactions revisited: synthesis of tetraphenylporphyrins under equilibrium conditions. *J. Org. Chem.* **52**, 827-836 (1987).

4. Boisbrun, M. *et al.* Design and photophysical properties of new RGD targeted tetraphenylchlorins and porphyrins. *Tetrahedron* **64**, 3494-3504 (2008).

5. Matsumoto, J., Matsumoto, T., Senda, Y., Shiragami T. & Yasuda, M. Preparation and characterization of porphyrin chromophores immobilized on micro-silica gel beads. *J. Photochem. Photobiol. A* **197**, 101-109 (2008).

6. Mulholland, A. R., Thordarson, P., Mensforth, E. J. & Langford, S. J. Porphyrin dyads linked by a rotatable 3,3′-biphenyl scaffold: a new binding motif for small ditopic molecules. *Org. Biomol. Chem.* **10**, 6045-6053 (2012).

7. Kang, B., Chung, S., Ahn, Y. D., Lee, J. & Seo, J. Porphyrin–Peptoid Conjugates: Face-to-Face Display of Porphyrins on Peptoid Helices. *Org. Lett.* **15**, 1670-1673 (2013).

8. Butterfoss, G. L. *et al.* De novo structure prediction and experimental characterization of folded peptoid oligomers. *Proc. Natl. Acad. Sci. U.S.A.* **109**, 14320-14325 (2012).

9. Voelz, V. A., Dill, K. A. & Chorny, I. Peptoid conformational free energy landscapes from implicit-solvent molecular simulations in AMBER. *Biopolymers* **96**, 639-650 (2011).

10. Mukherjee, S., Zhou, G., Michel, C. & Voelz, V. A. Insights into Peptoid Helix Folding Cooperativity from an Improved Backbone Potential. *J. Phys. Chem. B* **119**, 15407-15417 (2015).

11. Wang, J., Wolf, R. M., Caldwell, J. W., Kollman, P. A. & Case, D. A. Development and testing of a general amber force field. *J. Comput. Chem.* **25**, 1157-1174 (2004).

12. Jakalian, A., Jack, D. B. & Bayly, C. I. Fast, efficient generation of high-quality atomic charges. AM1-BCC model: II. Parameterization and validation. *J. Comput. Chem.* **23**, 1623-1641 (2002).

13. Onufriev, A., Case, D. A. & Bashford, D. Effective Born radii in the generalized Born approximation: The importance of being perfect. *J. Comput. Chem.* **23**, 1297-1304 (2002).

14. McGibbon, R. T. *et al*. MDTraj: A Modern Open Library for the Analysis of Molecular Dynamics Trajectories. *Biophys. J.* **109**, 1528-1532 (2015).

15. Lee, S., Lee, J. & Pang, Y. Excited state intramolecular proton transfer of 1,2-dihydroxyanthraquinone by femtosecond transient absorption spectroscopy. *Curr. Appl. Phys*. **15**, 1492-1499 (2015).

16. Lee, J., Lee, S., Jen, M. & Pang, Y. *J.* Metal-Enhanced Fluorescence: Wavelength-Dependent Ultrafast Energy Transfer. *Phys. Chem. C* **119**, 23285-23291 (2015).

17. Lee, I., Lee, S. & Pang, Y. Excited-State Dynamics of Carotenoids Studied by Femtosecond Transient Absorption Spectroscopy. *Bull. Korean Chem. Soc.* **35**, 851-857 (2014).

18. van Stokkum, I. H., Larsen, D. S. & van Grondelle, R. Global and target analysis of time-resolved spectra.

Biochim. Biophys. Acta **1657**, 82-104 (2004).
19. Snellenburg, J. J., Laptenok, S. P., Seger, R., Mullen, K. M. & van Stokkum, I. H. M. Glotaran: A Java-Based Graphical User Interface for the R Package TIMP. *J. Stat. Softw.* **49**, 1-22 (2012).
